# Exosomes as nanostructures deliver miR-204 in alleviation of mitochondrial dysfunction in diabetic nephropathy through suppressing methyltransferase-like 7A-mediated CIDEC N6-methyladenosine methylation

**DOI:** 10.18632/aging.205535

**Published:** 2024-02-08

**Authors:** Juan Jin, Yiwei Shang, Siqiang Zheng, Limiao Dai, Jiyu Tang, Xueyan Bian, Qiang He

**Affiliations:** 1 Department of Nephrology, The First Affiliated Hospital of Zhejiang Chinese Medical University (Zhejiang Provincial Hospital of Chinese Medicine), Hangzhou, Zhejiang 310000, China; 2 Clinical School of Medicine, Hangzhou Normal University, Hangzhou, Zhejiang 310004, China; 3 Department of Nephrology, Ningbo First Hospital, Ningbo, Zhejiang 315010, China

**Keywords:** diabetic nephropathy, exosome, microRNA-204, mitochondrial dysfunction, methyltransferase-like 7A

## Abstract

Objective: The exosomal cargo mainly comprises proteins, lipids, and microRNAs (miRNAs). Among these, miRNAs undertake multiple biological effects of exosomes (Exos). Some stem cell-derived exosomal miRNAs have shown the potential to treat diabetic nephropathy (DN). However, there is little research into the therapeutic effects of adipose-derived stem cell (ADSC)-derived exosomal miRNAs on DN. We aimed to explore the potential of miR-204-modified ADSC-derived Exos to mitigate DN.

Methods: Exos were extracted and identified from ADSCs. Histopathological injury, oxidative stress (OS), mitochondrial function, cell viability, and apoptosis were assessed to explore the effects of ADSC-derived Exos on DN. For mechanism exploration, quantitative real-time polymerase chain reaction (qRT-PCR) and western blotting were used to measure miR-204, methyltransferase (METTL3, METTL14, and METTL7A), and CIDEC. Also, CIDEC m6A methylation and miR-204-METTL7A, and METTL7A-CIDEC interactions were determined.

Results: Initially, OS-induced mitochondrial dysfunction was observed in DN rats. ADSC-derived Exos inhibited histopathological injury, cell apoptosis, OS, and mitochondrial dysfunction in DN rats. The similar therapeutic effects of ADSC-derived Exos were detected in the *in vitro* model. Intriguingly, miR-204 was released by ADSC-derived Exos and its upregulation enhanced the anti-DN effects of Exos. Mechanically, miR-204 reduced METTL7A expression to CIDEC m6A methylation, thus suppressing OS and mitochondrial dysfunction.

Conclusions: ADSC-derived exosomal miR-204 rescued OS-induced mitochondrial dysfunction by inhibiting METTL7A-mediated CIDEC m6A methylation. This study first revealed the significant role of ADSC-derived exosomal miR-204 in DN, paving the way for the development of novel therapeutic strategies to improve the clinical outcomes of DN patients.

## INTRODUCTION

Diabetic nephropathy (DN) is a prevalent diabetic complication, as well as a dominant contributing factor to end-stage renal disorder (ESRD) [1, 2]. Previous reports have shown that DN affects 25–40% of individuals with type 1 diabetes, resulting in a death rate of 30–40% [1, 3]. DN is clinically featured by increased unrelenting albuminuria, a thickened glomerular basement membrane, and an aggregated extracellular matrix, resulting in podocyte injury and renal dysfunction [4]. The pathogenesis of DN is complicated. Multiple factors, including oxidative stress (OS), lipid disorders, inflammatory responses, and insulin resistance, are involved in its onset and development [5]. Significant elevations of reactive oxygen species (ROS) levels have been found in diabetes and are considered the main cause of diabetic complications [6]. OS can damage podocytes, endothelial and mesangial cells, promote transforming growth factor (TGF)-β expression, microalbuminuria, and glomerular apoptosis, contributing to the progression of DN into ESRD. Moreover, mitochondria can undergo excessive OS under hyperglycemic conditions, leading to DNA damage and ultimately promoting renal cell apoptosis [7]. OS-mediated mitochondrial dysfunction has been demonstrated to be a crucial part of DN pathogenesis [8]. However, conventional therapeutic strategies, including glucose and weight control, and renin-angiotensin-aldosterone system blockage, are insufficient to prevent DN progression [9]. Therefore, new therapeutic approaches for ND, especially targeted mitochondrial dysfunction, are desperately needed.

Stem cells, known for their capacity for self-renewing and generating specialized cell types, have been regarded as a potential therapeutic tool for DN [10]. Notably, exosomes (Exos) derived from stem cells have shown great potential in the treatment of DN [11–13]. Exos are extracellular vesicles (30–150 nm in diameter) that consist of proteins, lipids, and nucleic acids, including noncoding RNA, microRNA (miRNA), and messenger RNA [14]. Moreover, Exos from bone marrow mesenchymal stem cells can ameliorate pathological injury in DN rats by reducing blood glucose and improving renal function [13]. Besides Exos from mesenchymal stem cells, those released by adipose-derived stem cells (ADSC) effectively mitigate DN symptoms *in vivo* [4]. Notably, miRNAs, which are a category of 18–23 nucleotide noncoding RNAs, can be loaded into Exos and function as paracrine regulators implicated in the regulation of various disorders [15, 16]. MiRNAs act as biomarkers to predict the occurrence of DN [17]. Interestingly, a recent study has revealed that the expression of miR-204 is decreased in DN patients and its upregulation inhibits high glucose (HG)-induced OS and extracellular matrix deposition [18]. Another study demonstrates that miR-204 can prevent diabetes-induced chronic renal injury [19]. Furthermore, *in vivo* studies have reported the alleviating effects of ADSC-derived exosomal miR-486 [4] and miR-215-5p [16] on DN symptoms. Nevertheless, the expression pattern of miR-204 in ADSC-derived Exos and its role in DN development remain unclear.

Over the past two decades, RNA methylation has become a research hotspot in diabetes and its complications. RNA methylation is an important form of post-translational modification that regulates epigenetic changes [20]. N6-methyladenosine (m6A) modification is a prevalent form of RNA methylation, comprising about 1/2 of all methylated ribonucleotides and 1/1,000-4/1,000 of adenosines [21]. m6A modification is initiated by methyltransferases (known as “writers”), while demethylases (called “erasers”) are responsible for its removal, and binding proteins (referred to as “readers”) are for its recognition [22]. Commonly, the enzyme complex, composed of methyltransferase-like (METTL) 3, METTL14, as well as Wilms tumor 1-associated protein (WTAP), acts as the catalyst for m6A [22, 23]. A heterodimer can be generated by METTL3 and METTL14, in which METTL3 serves as the core catalytic subunit, whereas METTL14 functions as the facilitator of RNA binding and METTL3 recognition; the formed heterodimer can further interact with WTAP [22]. m6A can affect RNA metabolism, including mRNA splicing, translation, localization, and stability, to modulate gene expression and various biological processes [22]. Hence, abnormal m6A modifications can contribute to various disorders, including DN [24]. Interestingly, a recent study has suggested that METTL7A exhibits m6A methyltransferase activity and mediates m6A methylation [25]. On the other hand, evidence shows that the crucial role of miRNAs in m6A methylation [26, 27]. miR-204 can target the methyltransferase, PRMT5, to affect cell proliferation and apoptosis [28]. Besides, IGF2BP3 (a m6A methylation regulator) has been identified as a target of miR-204 in kidney renal clear cell carcinoma [27]. However, the crosstalk between miR-204 and METTL7A has yet to be explored.

The cell death-inducing DFF45-like effector (CIDE) family that consists of CIDEA, CIDEB, and CIDEC triggers cell apoptosis [29, 30]. Among these, CIDEC is proven linked with insulin resistance and metabolic syndrome [30]. Previous research showed that CIDEC deficiency could mitigate pulmonary vascular remodeling in type 2 diabetes rat models [30]. A recent study has shown that CIDEC knockdown can ameliorate metabolic abnormalities, insulin resistance, and renal impairment, suppress cell apoptosis and renal fibrosis, and enhance autophagy to prevent DN progression [31]. Despite little knowledge of the relationship between methyltransferases and CIDEC, evidence has shown that METTL7A also plays a vital role in cell apoptosis and survival [32]. It is essential to explore the possible link between METTL7A and CIDEC in DN.

In this research, we disclosed innovative observations concerning the existence of miR-204 in ADSC-derived Exos. Furthermore, we delved into the role and underlying mechanism of ADSC-derived exosomal miR-204 in mitochondrial dysfunction in DN, with a focus on the molecular mechanism of m6A methylation. This study provides novel ideas about DN pathogenesis and furnishes new therapeutic targets for this disorder.

## RESULTS

### Inhibition of OS-induced mitochondrial dysfunction mitigates the pathological injury of DN rats

**Figure 1:**
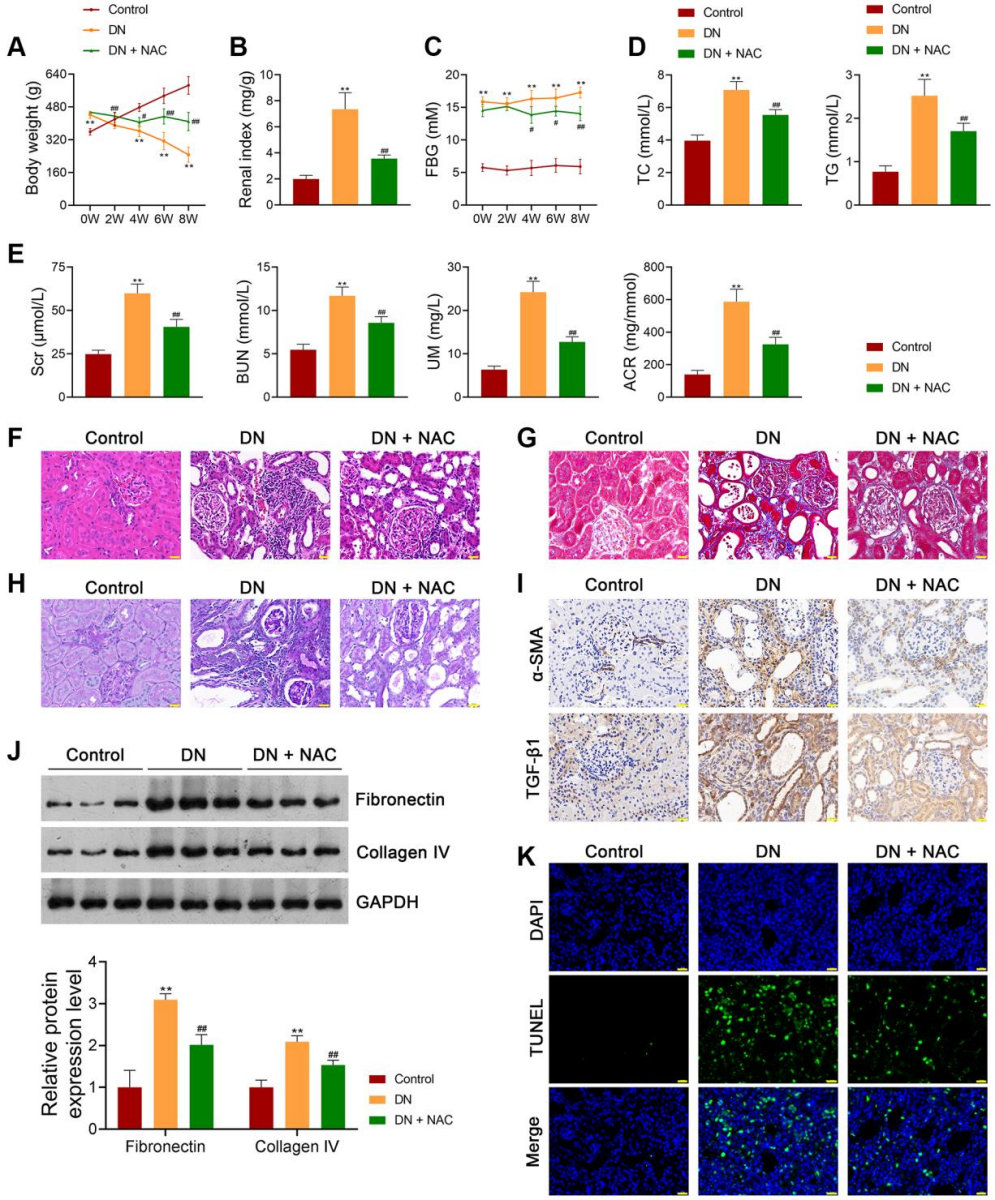
**Inhibition of OS ameliorates the symptoms of DN in rats.** (**A**–**C**) The body weights, renal index, and FBG levels of rats in each group. (**D**, **E**) Plasma TC, TG, Scr, BUN, UM, and ACR of rats in each group using enzymatic colorimetric assay and ELISA. (**F**–**H**) Histopathological examinations in the kidney tissues of rats in each group using HE, Masson, and PAS staining (scale bar = 20 μm). (**I**) The expression of α-SMA and TGF-β1 in the kidney tissues of rats in each group using immunohistochemistry (scale bar = 20 μm). (**J**) The expression of fibronectin and collagen IV in the kidney tissues of rats in each group using western blotting. (**K**) Cell apoptosis in the kidney tissues of rats in each group using TUNEL assay (scale bar = 20 μm). To explore the role of OS in DN development, DN rats were treated with or without the ROS inhibitor, N-Acetylcysteine (NAC; 20 mg/kg) for eight successive weeks. Data were expressed as mean ± standard deviation (*n* = 6/group). ^**^p < 0.01 vs. Control group; ^#^*p* < 0.05 and ^##^*p* < 0.01 vs. DN group. Abbreviations: OS: oxidative stress; DN: diabetic nephropathy; FBG: fasting blood-glucose; TC: total cholesterol, TG: triglyceride; BUN: blood urea nitrogen; Scr: serum creatinine; UM: urinary microalbumin; ACR: albumin to creatinine ratio; ELISA: enzyme-linked immunosorbent assay; HE: hematoxylin-eosin; PAS: periodic acid-Schiff; α-SMA: alpha-smooth muscle actin; TGF-β1: transforming growth factor beta 1; TUNEL: terminal deoxynucleotidyl transferase-mediated dUTP nick-end labeling; ROS: reactive oxygen species; NAC: N-Acetylcysteine.

**Figure 2:**
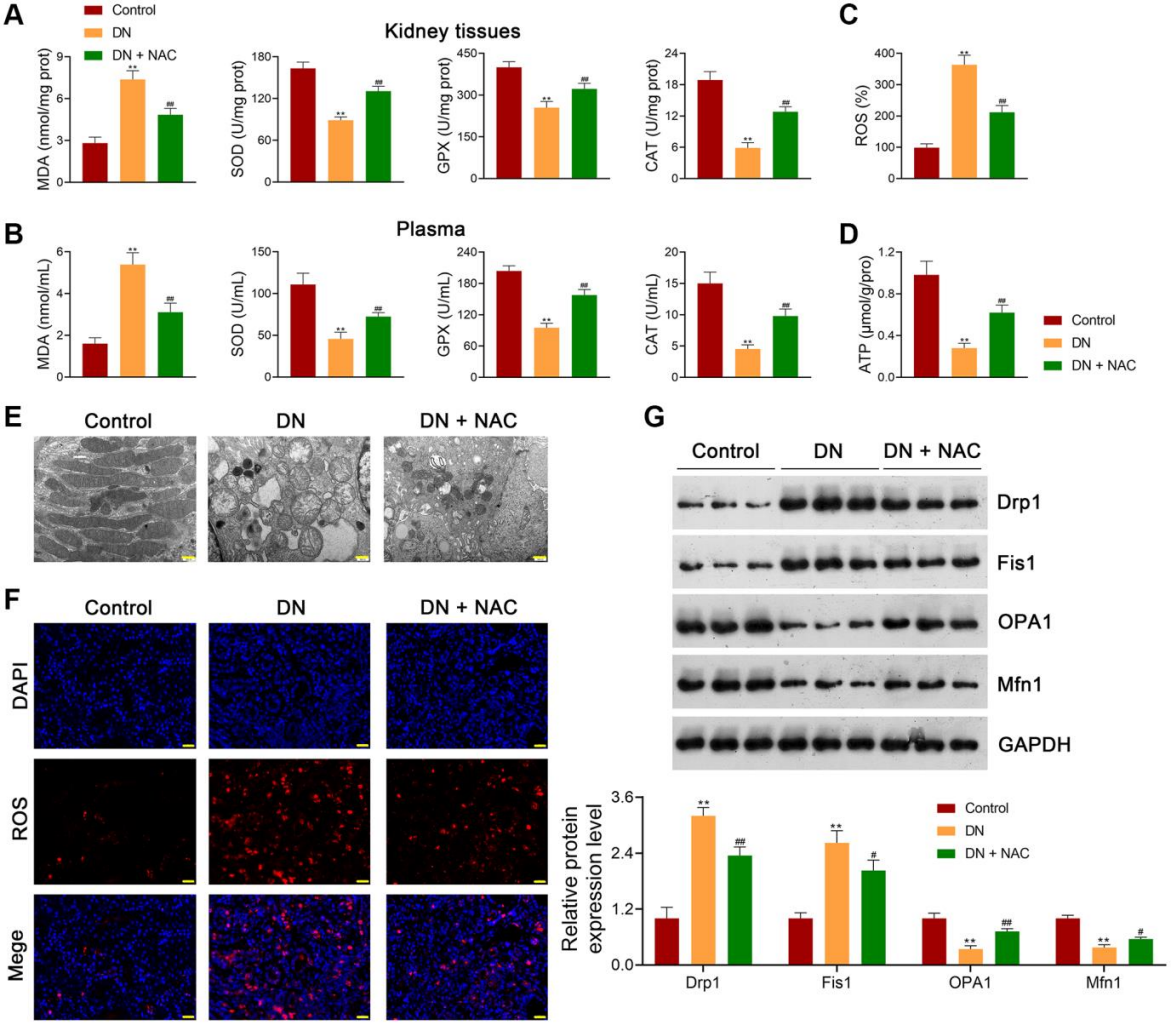
**Inhibition of OS rescues mitochondrial dysfunction in DN rats.** (**A**, **B**) Assessment of the levels of MDA, SOD, GPX, and CAT in the kidney tissues and plasma of rats in each group using ELISA. (**C**, **D**) ROS production and ATP release in the kidney tissues of rats in each group. (**E**) Observation of mitochondrial morphology under TEM (scale bar = 500 nm). (**F**) Mitochondrial ROS production in the kidney tissues of rats in each group (scale bar = 20 μm). (**G**) The expression of Drp1, Fis1, OPA1, and Mfn1 in the kidney tissues of rats in each group using western blotting. To figure out the impact of OS on mitochondrial function in DN, DN rats were treated with or without the ROS inhibitor, N-Acetylcysteine (NAC; 20 mg/kg) for eight successive weeks. Data were expressed as mean ± standard deviation (*n* = 6/group). ^**^p < 0.01 vs. Control group; ^#^*p*< 0.05 and ^##^*p* < 0.01 vs. DN group. Abbreviations: OS: oxidative stress; DN: diabetic nephropathy; MDA: malondialdehyde; SOD: superoxide dismutase; GPX: glutathione peroxidase; CAT: catalase; ELISA: enzyme-linked immunosorbent assay; ROS: reactive oxygen species; ATP: adenosine triphosphate; TEM: transmission electron microscopy; NAC: N-Acetylcysteine.

In this study, an *in vivo* model of DN was established by feeding rats a high-fat diet and streptozotocin (STZ) treatment. To investigate whether the pathology of DN is associated with OS, DN rats were treated with or without the ROS inhibitor N-Acetylcysteine (NAC) to investigate whether OS is implicated in DN pathology. Our results showed that the body weights of DN rats were significantly lower than those of control rats (*p <* 0.01; Figure 1A). In contrast, the renal index was markedly increased in DN rats compared to that of control rats (*p <* 0.01; Figure 1B). Moreover, elevated fasting blood glucose (FBG) levels were found in DN rats compared to those in control rats (*p <* 0.01; Figure 1C). The DN indicators, total cholesterol (TC), triglyceride (TG), serum creatinine (Scr), blood urea nitrogen (BUN), urinary microalbumin (UM), and albumin-to-creatinine ratio (ACR) were notably higher in DN rats than in control rats (*p <* 0.01; Figure 1D, 1E). Hematoxylin-eosin (HE) and Masson staining showed that cell infiltration and tubulointerstitial fibrosis were promoted in the kidney tissues of DN rats (Figure 1F, 1G). Periodic acid-Schiff (PAS) staining revealed a thickened tubular basement membrane and enhanced glycogen deposition in the kidney tissues of DN rats (Figure 1H). These results suggested the successful establishment of the *in vivo* DN model. NAC administration alleviated the symptoms and histopathological injury in DN rats (Figure 1A–1H). Furthermore, NAC decreased the expression levels of pro-fibrotic factors, alpha-smooth muscle actin (α-SMA), and transforming growth factor beta 1 (TGF-β1), in DN rats (Figure 1I). Western blotting showed that the expression levels of fibronectin and collagen IV were significantly increased in DN rats compared to control rats (*p <* 0.01); however, the increasing trend was abolished by NAC treatment (*p <* 0.01; Figure 1J). Increased cell apoptosis was observed in DN rats, which was suppressed after NAC treatment (Figure 1K). The levels of the OS marker, malondialdehyde (MDA), in the kidney tissues and plasma of DN rats, were markedly upregulated compared to those in control rats (*p <* 0.01), while the levels of the antioxidant markers, superoxide dismutase (SOD), glutathione peroxidase (GPX), and catalase (CAT), were downregulated (*p <* 0.01; Figure 2A, 2B). NAC treatment reduced MDA levels and elevated the levels of SOD, GPX, and CAT in the kidney tissues (*p <* 0.01; Figure 2A) and plasma (*p <* 0.01; Figure 2B) of DN rats. In addition, ROS levels in the kidney tissues of DN rats were markedly upregulated, whereas ATP levels were downregulated (*p <* 0.01; Figure 2C, 2D). NAC administration led to a notable decrease in ROS levels and an increase in adenosine triphosphate (ATP) levels (*p <* 0.01; Figure 2C, 2D).

Moreover, the mitochondrial function of rats with DN was evaluated to determine whether mitochondrial dysfunction occurs during DN development. Transmission electron microscopy (TEM) revealed ruptured renal tubule mitochondria in DN rats, which were rescued by NAC administration (Figure 2E). Meanwhile, mitochondrial ROS accumulation in DN rats was enhanced compared to that in control rats (Figure 2F). Additionally, the levels of Drp1 and Fis1 (mitochondrial fission-related proteins) were significantly upregulated in DN rats, whereas those of OPA1 and Mfn1 (mitochondrial fusion-related proteins) were downregulated (*p <* 0.01; Figure 2G). NAC treatment reduced Drp1 and Fis1 levels and elevated OPA1 and Mfn1 levels in DN rats (*p <* 0.05; Figure 2G).

### ADSC-derived Exos ameliorate DN by mitigating mitochondrial dysfunction

ADSCs were isolated from the adipose tissue of obese rats and identified by observing their morphology and differentiation ability, as well as by assessing their surface markers (CD29, CD44, CD45, and CD99). In this study, we observed cobblestone-shaped ADSCs, adipocytes, and osteoblasts under a microscope (Supplementary Figure 1A, 1B). Flow cytometry showed positive expression of CD29 (99.26%), CD44 (98.18%), and CD99 (98.65%), and negative expression of CD45 (0.45%) (Supplementary Figure 1C). TEM observations revealed cup-shaped Exos (Supplementary Figure 1D); in addition, the particle sizes of these Exos were 45–140 nm, with a mean value of 70.75 nm (Supplementary Figure 1E). Western blotting revealed the positive expression of exosomal markers (CD9, CD63, and CD81), further indicating the successful isolation of Exos from ADSCs (Supplementary Figure 1F).

**Figure 3:**
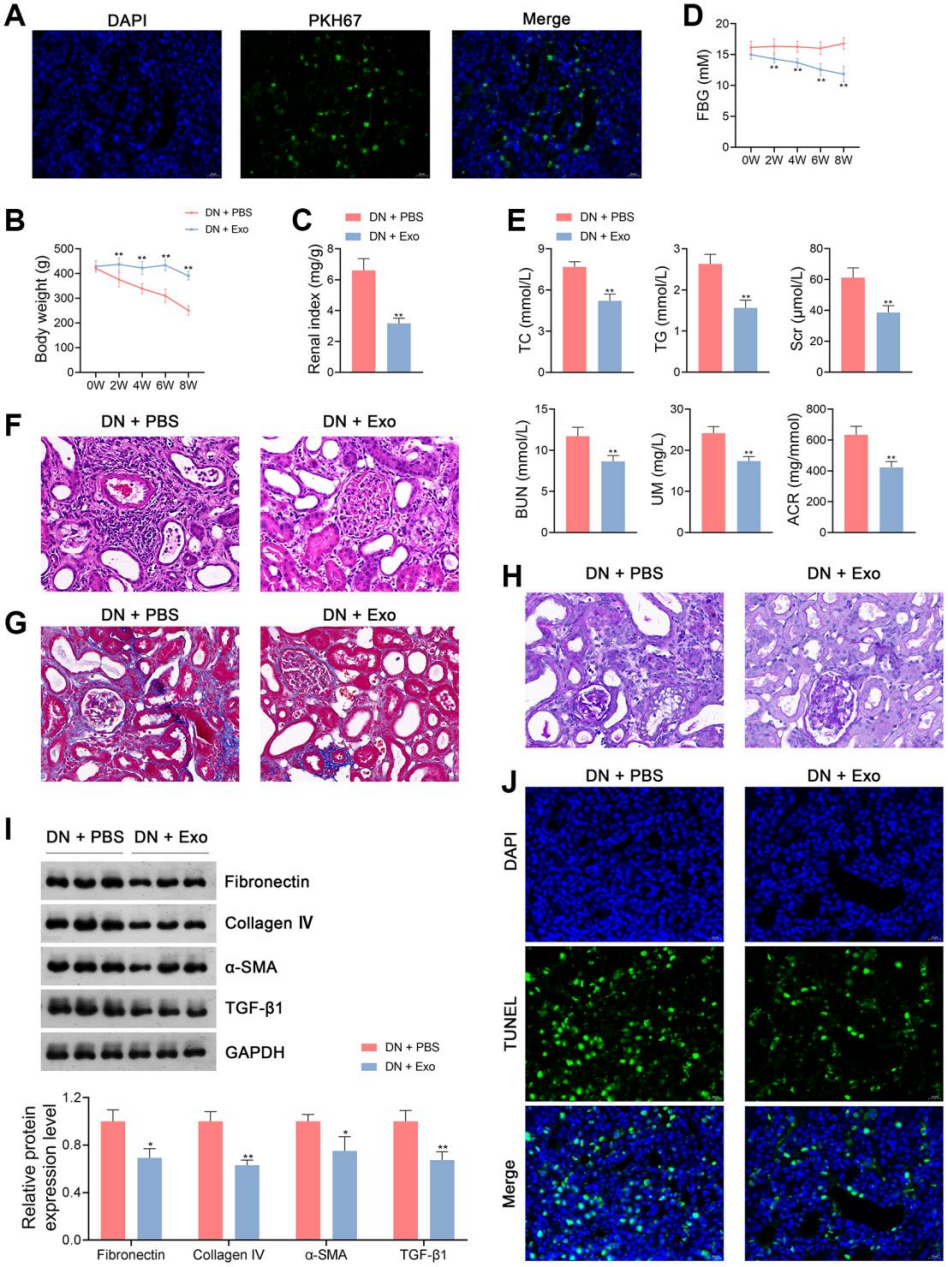
**ADSC-derived Exos ameliorate the symptoms of DN in rats.** (**A**) Determination of ADSC-derived Exos taken up by the kidney tissue cells of rats in each group using PKH67 staining. (**B**–**D**) The body weights, renal index, and FBG levels of rats in each group. (**E**) Plasma TC TG, Scr, BUN, UM, and ACR of rats in each group using enzymatic colorimetric assay and ELISA. (**F**–**H**) Histopathological examinations in the kidney tissues of rats in each group using HE, Masson, and PAS staining (scale bar = 20 μm). (**I**) The expression of fibronectin, collagen IV, α-SMA, and TGF-β1 in the kidney tissues of rats in each group using western blotting. (**J**) Cell apoptosis in the kidney tissues of rats in each group using TUNEL assay (scale bar = 20 μm). To explore the therapeutical effects of ADSC-derived Exos on DN, rats were subjected to caudal vein injection with Exos (1.6 mg/kg) for eight successive weeks. Rats treated with the same volume of PBS served as controls. Data were expressed as mean ± standard deviation (*n* = 6/group). ^*^p < 0.05 and ^**^p < 0.01 vs. DN + PBS group. Abbreviations: ADSC: adipose-derived stem cell; Exos: exosomes; DN: diabetic nephropathy; FBG: fasting blood-glucose; TC: total cholesterol; TG: triglyceride; BUN: blood urea nitrogen; Scr: serum creatinine; UM: urinary microalbumin; ACR: albumin to creatinine ratio; ELISA: enzyme-linked immunosorbent assay; HE: hematoxylin-eosin; PAS: periodic acid-Schiff; α-SMA: alpha-smooth muscle actin; TGF-β1: transforming growth factor beta 1; TUNEL: terminal deoxynucleotidyl transferase-mediated dUTP nick-end labeling; PBS: phosphate-buffered saline.

To determine whether ADSC-derived Exos could be taken up by receptor cells, PKH67 staining was used to label Exos in the kidney tissues of rats. We observed that Exos were distributed in the cytoplasm of kidney tissue cells of Exo-treated rats (Figure 3A).

**Figure 4:**
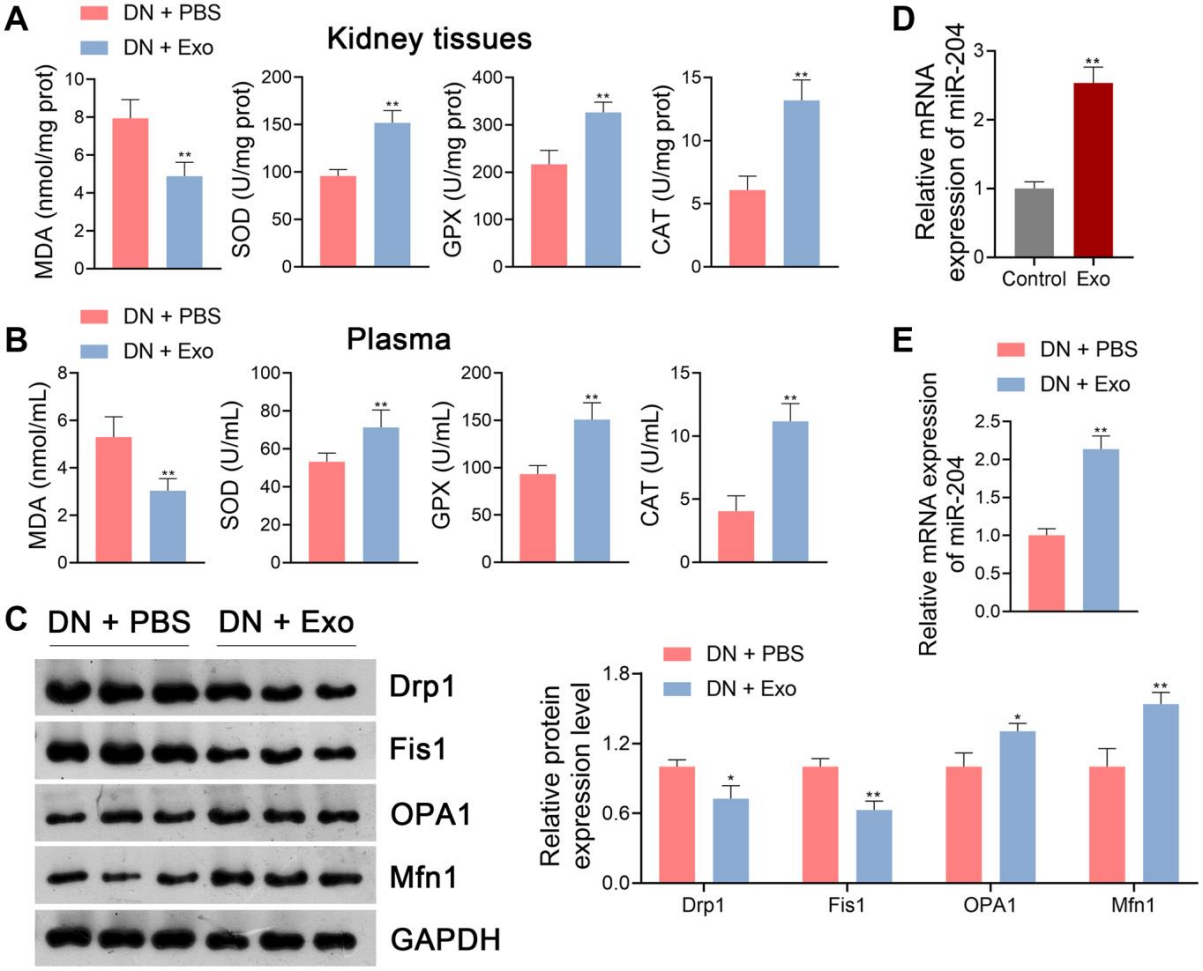
**ADSC-derived Exos suppressed OS and mitochondrial dysfunction in DN rats.** (**A**, **B**) The levels of MDA, SOD, GPX, and CAT in the kidney tissues and plasma of rats in each group using ELISA. (**C**) The expression of Drp1, Fis1, OPA1, and Mfn1 in the kidney tissues of rats in each group using western blotting. (**A**–**C**) To explore the effects of ADSC-derived Exos on OS and mitochondrial function in DN, DN rats were subjected to caudal vein injection with Exos (1.6 mg/kg) for eight successive weeks. Rats treated with same volume of PBS served as controls. Data were expressed as mean ± standard deviation (*n* = 6/group). ^*^p < 0.05 and ^**^p < 0.01 vs. DN + PBS group. (**D**) The expression of miR-204 in ADSC-derived Exos using qRT-PCR. ADSCs without any treatment served as the controls. Data were expressed as mean ± standard deviation. ^**^p < 0.01 vs. Control group. (**E**) The expression of miR-204 in the kidney tissues of rats in each group using qRT-PCR. To explore the effects of ADSC-derived Exos on miR-204 expression in DN, DN rats were subjected to caudal vein injection with Exos (1.6 mg/kg) for eight successive weeks. Rats treated with same volume of PBS served as controls. Data were expressed as mean ± standard deviation (*n* = 6/group). ^**^p < 0.01 vs. DN + PBS group. Abbreviations: ADSC: adipose-derived stem cell; Exos: exosomes; OS: oxidative stress; DN: diabetic nephropathy; MDA: malondialdehyde; SOD: superoxide dismutase; GPX: glutathione peroxidase; CAT: catalase; ELISA: enzyme-linked immunosorbent assay; miR-204: microRNA-204; qRT-PCR: quantitative real-time polymerase chain reaction; PBS: phosphate-buffered saline.

Previous studies have demonstrated that ADSC-derived Exos effectively ameliorate DN symptoms [4]. Therefore, rats with DN were treated with either phosphate-buffered saline (PBS) or ADSC-derived Exos to determine the effects of Exos on DN pathology. Our data showed that Exo treatment significantly increased body weight and decreased the renal index of DN rats (*p <* 0.01; Figure 3B, 3C). In addition, FBG levels in DN rats were notably reduced following Exo treatment (*p <* 0.01; Figure 3D). Similarly, Exo treatment significantly downregulated TC, TG, Scr, BUN, UM, and ACR levels in DN rats (*p <* 0.01; Figure 3E). Following Exo treatment, cell infiltration and tubulointerstitial fibrosis in the kidney tissues of DN rats were alleviated by thinning the tubular basement membrane and lowering glycogen deposition (Figure 3F–3H). Exos markedly decreased the expression levels of fibronectin, collagen IV, α-SMA, and TGF-β1 in DN rats (*p* < 0.05; Figure 3I). Cell apoptosis was inhibited in DN rats following Exo treatment (Figure 3J). In terms of the OS status, MDA levels in the kidney tissues and plasma of DN rats were notably decreased by Exos, whereas the levels of SOD, GPX, and CAT were increased (*p* < 0.01; Figure 4A, 4B). Furthermore, Exo treatment led to a significant reduction in the levels of Drp1 and Fis1 and an increase in those of OPA1 and Mfn1 in DN rats (*p* < 0.05; Figure 4C).

### ADSC-derived exosomal miR-204 mitigates HG-induced injury in renal tubular cells

Evidence indicates that miR-204 is involved in the regulation of OS during DN progression [18]. Thus, we hypothesized that miR-204 is released by ADSC-derived Exos in the pathological mechanisms of DN. qRT-PCR showed that miR-204 expression in ADSC- derived Exos was markedly higher than that in control ADSCs (*p* < 0.01; Figure 4D). Furthermore, elevated miR-204 expression was observed in DN rats following Exo treatment (*p* < 0.01; Figure 4E).

**Figure 5:**
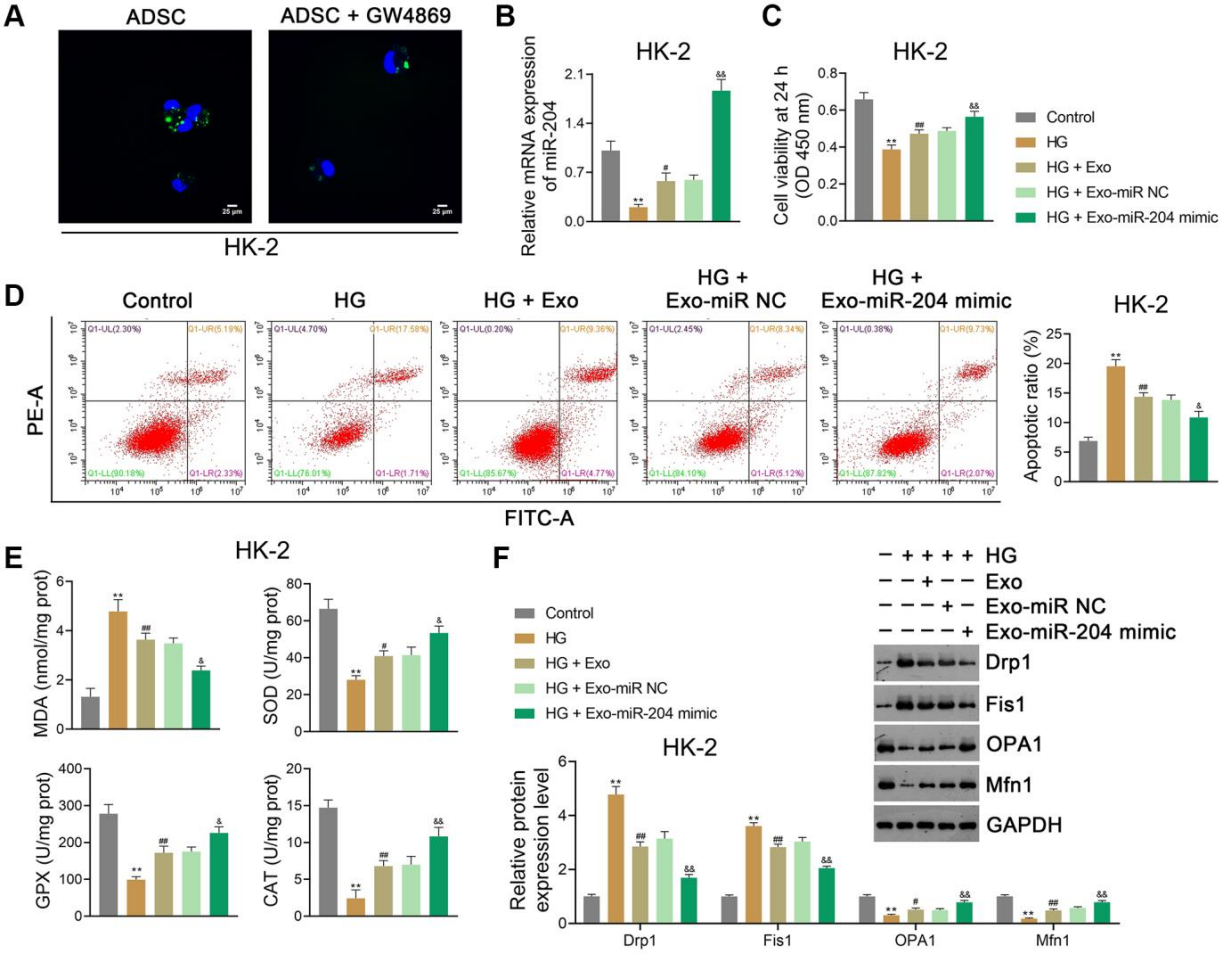
**ADSC-derived exosomal miR-204 alleviates HG-induced injury in HK-2 cells.** (**A**) Determination of ADSC-derived Exos taken up by HK-2 cells (human proximal renal tubular epithelial cell line) using PKH67 staining. ADSCs were pre-treated with GW4869 (an Exo inhibitor; 2.5 μM), followed by Exo isolation. HK-2 cells were then co-cultured with PKH67-labeled Exos to evaluate their capacity to take up Exos. (**B**) The expression of miR-204 in HK-2 cells using qRT-PCR. (**C**, **D**) HK-2 cell viability (at 24 h) and apoptosis assessed using CCK-8 and flow cytometry, respectively. (**E**) The levels of MDA, SOD, GPX, and CAT in HK-2 cells using ELISA. (**F**) The expression of Drp1, Fis1, OPA1, and Mfn1 in HK2 cells using western blotting. (**B**–**F**) HK-2 cells were exposed to HG (30 mM) and cultured for 24 h to construct an *in vitro* model of DN. To determine the function of exosomal miR-204 in DN, ADSCs were transfected with miR-204 mimic or miR NC (negative control) for 48 h. Then, Exos were separated from the transfected ADSCs and co-cultured with HK-2 cells for 12 h at the concentration of 100 μg/mL. Data were expressed as mean ± standard deviation. ^**^p < 0.01 vs. Control group; ^#^*p* < 0.05 and ^##^*p* < 0.01 vs. HG group; ^&^*p* < 0.05 and ^&&^*p* < 0.01 vs. HG + Exo-miR NC group. Abbreviations: ADSC: adipose-derived stem cell; miR-204: microRNA-204; HG: high glucose; Exos: exosomes; qRT-PCR: quantitative real-time polymerase chain reaction; CCK-8: cell counting kit-8; MDA: malondialdehyde; SOD: superoxide dismutase; GPX: glutathione peroxidase; CAT: catalase; ELISA: enzyme-linked immunosorbent assay.

Given that ADSC-derived Exos were found to upregulate miR-204 expression in DN rats, *in vitro* experiments were conducted to further investigate the role of ADSC-derived exosomal miR-204 on DN. ADSCs were pre-treated with the Exo inhibitor GW4869, followed by Exo isolation. HK-2 and NRK-52E cells were then co-cultured with PKH67-labeled Exos to evaluate their capacity to take up Exos. We observed that Exos were distributed in the cytoplasm of ADSC-co-cultured HK-2 cells, especially in the absence of GW4869 (Figure 5A). In the present study, HG (30 mM) treatment was used to induce *in vitro* models of DN, and ADSCs were transfected with either the miR-204 mimic or miR-NC (negative control). Exos were then separated from the transfected ADSCs and co-cultured with HG-treated HK-2 and NRK-52E cells. Our data showed that HG treatment significantly decreased miR-204 expression in HK-2 cells (*p <* 0.01), which was reversed by Exo treatment (*p* < 0.01; Figure 5B). The miR-204 mimic further enhanced miR-204 expression in HG-treated HK-2 cells compared to Exo-treated cells (*p* < 0.01; Figure 5B). In addition, HG treatment markedly suppressed the viability (at 24 h) and promoted the apoptosis of HK-2 cells (*p <* 0.01), which were abolished by Exo treatment (*p* < 0.01; Figure 5C, 5D). The miR-204 mimic strengthened the effects of Exos by increasing cell viability and inhibiting apoptosis in HG-treated HK-2 cells (*p* < 0.05; Figure 5C, 5D). After HG treatment, OS was promoted in HK-2 cells, wherein the MDA level was markedly upregulated, and the levels of SOD, GPX, and CAT were downregulated (*p* < 0.01; Figure 5E). Exos suppressed OS in HG-treated HK-2 cells, and the miR-204 mimic enhanced the anti-OS function of Exos (*p* < 0.05; Figure 5E). Similarly, Exos significantly reduced Drp1 and Fis1 expression and elevated OPA1 and Mfn1 expression in HG-treated HK-2 cells (*p* < 0.01); the miR-204 mimic strengthened the protective effect on mitochondrial function (*p* < 0.01; Figure 5F). Similar results were obtained in NRK-52E cells (Supplementary Figure 2A–2F).

### ADSC-derived exosomal miR-204 alleviates HG-induced injury in renal tubular cells by suppressing METTL7A-medicated m6A methylation

**Figure 6:**
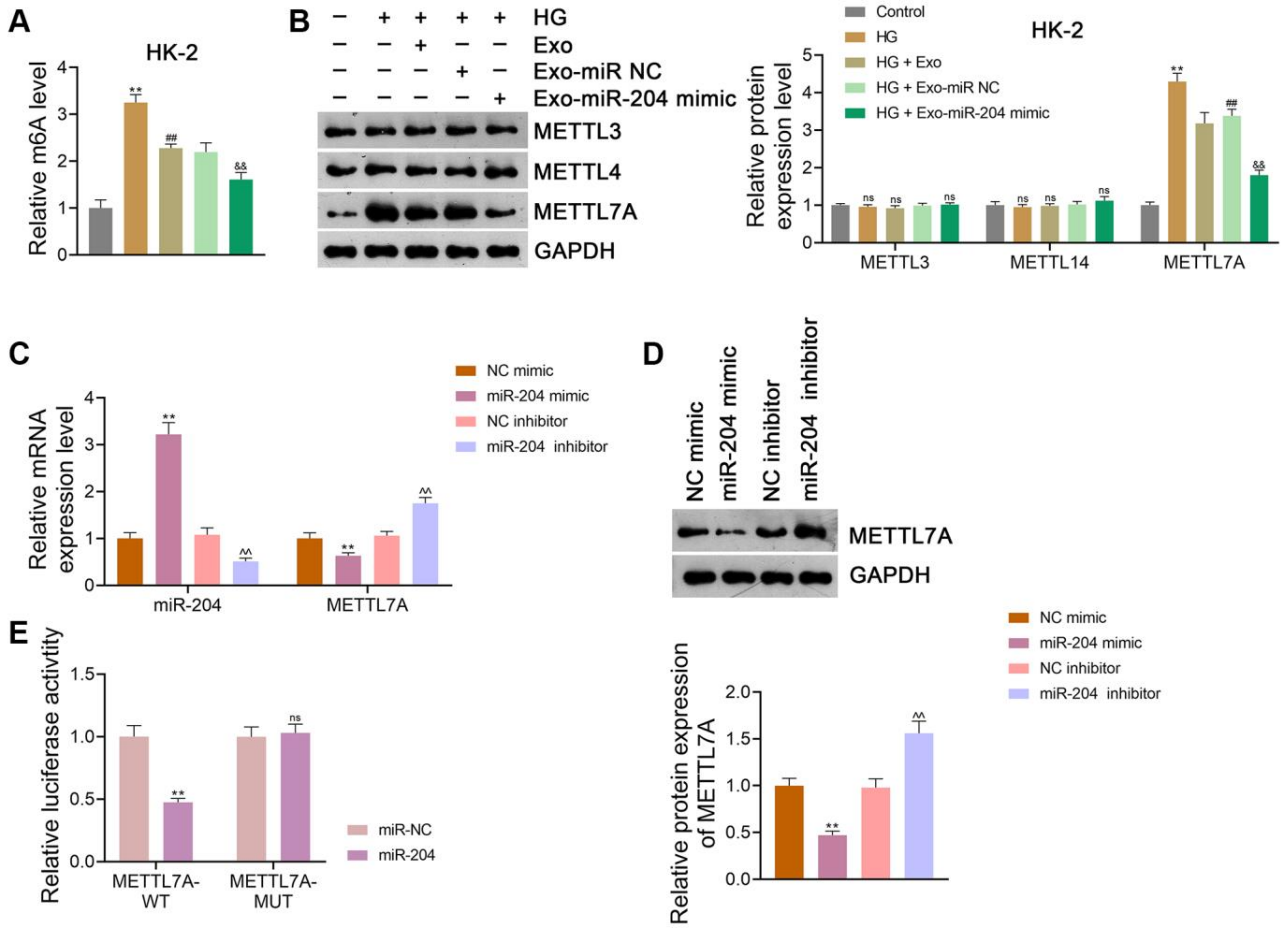
**ADSC-derived Exos modified by miR-204 inhibits METTL7A-medicated m6A methylation in HG-induced HK-2 cells.** (**A**) m6A methylation level in HK-2 cells. (**B**) The expression of METTL3, METTL14, and METTL7A in HK-2 cells using western blotting. (**A**, **B**) HK-2 cells were exposed to HG (30 mM) and cultured for 24 h to induce an *in vitro* model of DN. To figure out the impact of exosomal miR-204 in m6A methylation in DN, ADSCs were transfected with miR-204 mimic or miR NC (negative control) for 48 h. Next, Exos were isolated from the transfected ADSCs and co-cultured with HK-2 a for 12 h at the concentration of 100 μg/mL. Data were expressed as mean ± standard deviation. ^**^p < 0.01 vs. Control group; ^##^*p* < 0.01 vs. HG group; ^&&^*p* < 0.01 vs. HG + Exo-miR NC group; ns indicates no significant differences between groups. (**C**, **D**) The expression of miR-204 and METTL7A in HEK293T cells using qRT-PCR and western blotting. (**C**, **D**) Data were expressed as mean ± standard deviation. ^**^p < 0.01 vs. mimic NC group; ^^^^*p* < 0.01 vs. inhibitor NC group. (**E**) Determination of the interaction between miR-204 and METTL7A in HEK293T cells using dual-luciferase reporter assay. Data were expressed as mean ± standard deviation. ^**^p < 0.01 vs. miR NC group; ns indicates no significant differences between groups. Abbreviations: ADSC: adipose-derived stem cell; Exos: exosomes; miR-204: microRNA-204; METTL: methyltransferase-like; m6A: N6-methyladenosine; HG: high glucose; qRT-PCR: quantitative real-time polymerase chain reaction.

It has been suggested that m6A methylation, commonly mediated by METTL3 and METTL14, can participate in DN development [33, 34]. Evidence shows that METTL7A is also involved in m6A methylation [25]. In this study, we found that m6A methylation levels were significantly elevated in HK-2 cells after HG treatment (*p <* 0.01), which was abolished by Exo treatment (*p* < 0.01; Figure 6A). The miR-204 mimic enhanced the inhibitory effect of Exos on m6A levels in HG-treated HK-2 cells (*p* < 0.01; Figure 6A). Furthermore, HG, Exos, and the miR-204 mimic did not significantly affect the expression of either METTL3 or METTL14 in HK-2 cells (Figure 6B). However, METTL7A expression in HK-2 cells was markedly upregulated after HG treatment (*p* < 0.01), and this increase was abolished by Exo treatment (*p* < 0.01; Figure 6B). The miR-204 mimic further decreased METTL7A expression in HG-treated HK-2 cells compared to Exo-treated cells (*p* < 0.01; Figure 6B). Consistent findings were obtained using NRK-52E cells (Supplementary Figure 2G, 2H).

To further determine whether miR-204 interacts with METTL7A, HEK293T cells were transfected with either mimic NC, miR-204 mimic, inhibitor NC, or the miR-204 inhibitor. Our results showed that the miR-204 mimic significantly reduced METTL7A expression in HEK293T cells with increased miR-204 expression (*p* < 0.01; Figure 6C, 6D). In contrast, the miR-204 inhibitor markedly elevated METTL7A expression in HEK293T cells and decreased miR-204 expression (*p* < 0.01; Figure 6C, 6D). Through the dual-luciferase reporter assay, we found that the miR-204 mimic markedly suppressed METTL7A-WT activity in HEK293T cells (*p* < 0.01), but did not affect METTL7A-MUT activity, indicating that METTL7A was a target of miR-204 (Figure 6E).

**Figure 7:**
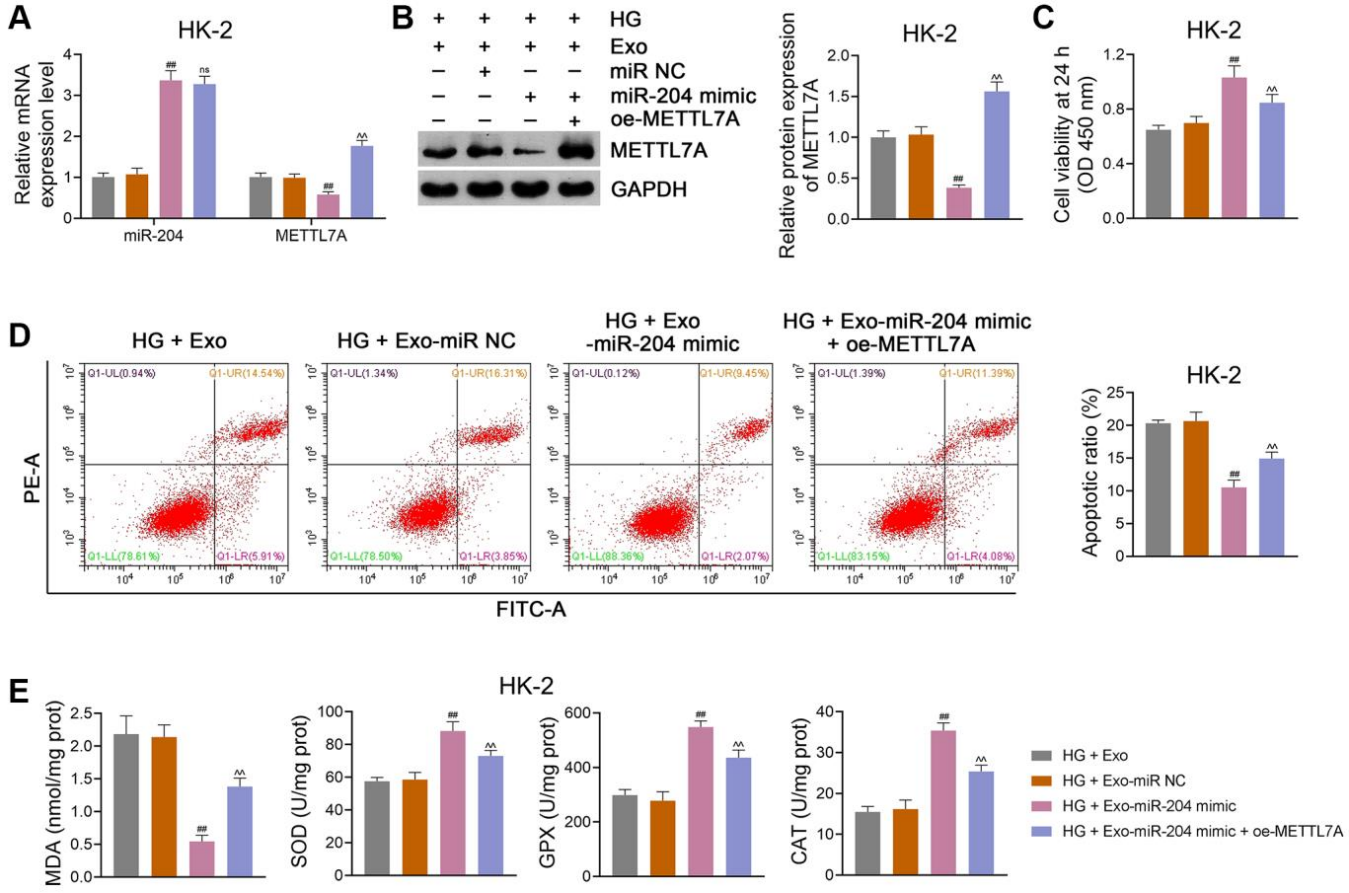
**ADSC-derived Exos modified by miR-204 prevent DN progression via downregulating METTL7A expression in HG-induced HK-2 cells.** (**A**, **B**) The expression of miR-204 and METTL7A in HK-2 cells using qRT-PCR and western blotting. (**C**, **D**) HK-2 cell viability (at 24 h) and apoptosis using CCK-8 and flow cytometry, respectively. (**E**) The levels of MDA, SOD, GPX, and CAT in HK-2 cells using ELISA. Exos were isolated from either miR-204 mimic- or miR NC-transfected ADSCs, which were subsequently co-cultured with HG-induced HK-2 cells for 12 h at the concentration of 100 μg/mL. Furthermore, HG-induced HK-2 cells were partially transected with oe-METTL7A and co-cultured with Exos from miR-204 mimic-transfected ADSCs to ascertain the link between miR-204 and METTL7A in DN. Data were expressed as mean ± standard deviation. ^##^*p* < 0.01 vs. HG + Exo-miR NC group; ^^^^*p* < 0.01 vs. HG + Exo-miR-204 mimic group; ns indicates no significant differences between groups. Abbreviations: ADSC: adipose-derived stem cell; Exos: exosomes; miR-204: microRNA-204; DN: diabetic nephropathy; METTL: methyltransferase-like; HG: high glucose; qRT-PCR: quantitative real-time polymerase chain reaction; CCK-8: cell counting kit-8; MDA: malondialdehyde; SOD: superoxide dismutase; GPX: glutathione peroxidase; CAT: catalase; ELISA: enzyme-linked immunosorbent assay.

In addition, HG-induced HK-2 cells were transfected with or without oe-METTL7A and co-cultured with ADSC-derived Exos to further validate the role of miR-204 and METTL7A in the anti-DN mechanism of ADSC-derived Exos. We found that the miR-204 mimic markedly decreased METTL7A expression in HG-treated HK-2 cells (*p* < 0.01), which was reversed by oe-METTL7A transfection (*p* < 0.01; Figure 7A, 7B). However, oe-METTL7A did not significantly affect the miR-204 expression in HG-treated HK-2 cells (Figure 7A). Moreover, oe-METTL7A transfection significantly abolished the pro-viability and anti-apoptotic effects of the miR-204 mimic in HG-treated HK-2 cells (*p* < 0.01; Figure 7C, 7D). Meanwhile, oe-METTL7A treatment markedly eliminated the effects of the miR-204 mimic on the downregulation of MDA and upregulation of SOD, GPX, and CAT in HG-treated HK-2 cells (*p* < 0.01; Figure 7E). Similar findings were obtained when NRK-52E cells were used (Supplementary Figure 3A–3E).

### METTL7A silencing alleviates HG-induced injury in renal tubular cells by inhibiting CIDEC m6A methylation

**Figure 8:**
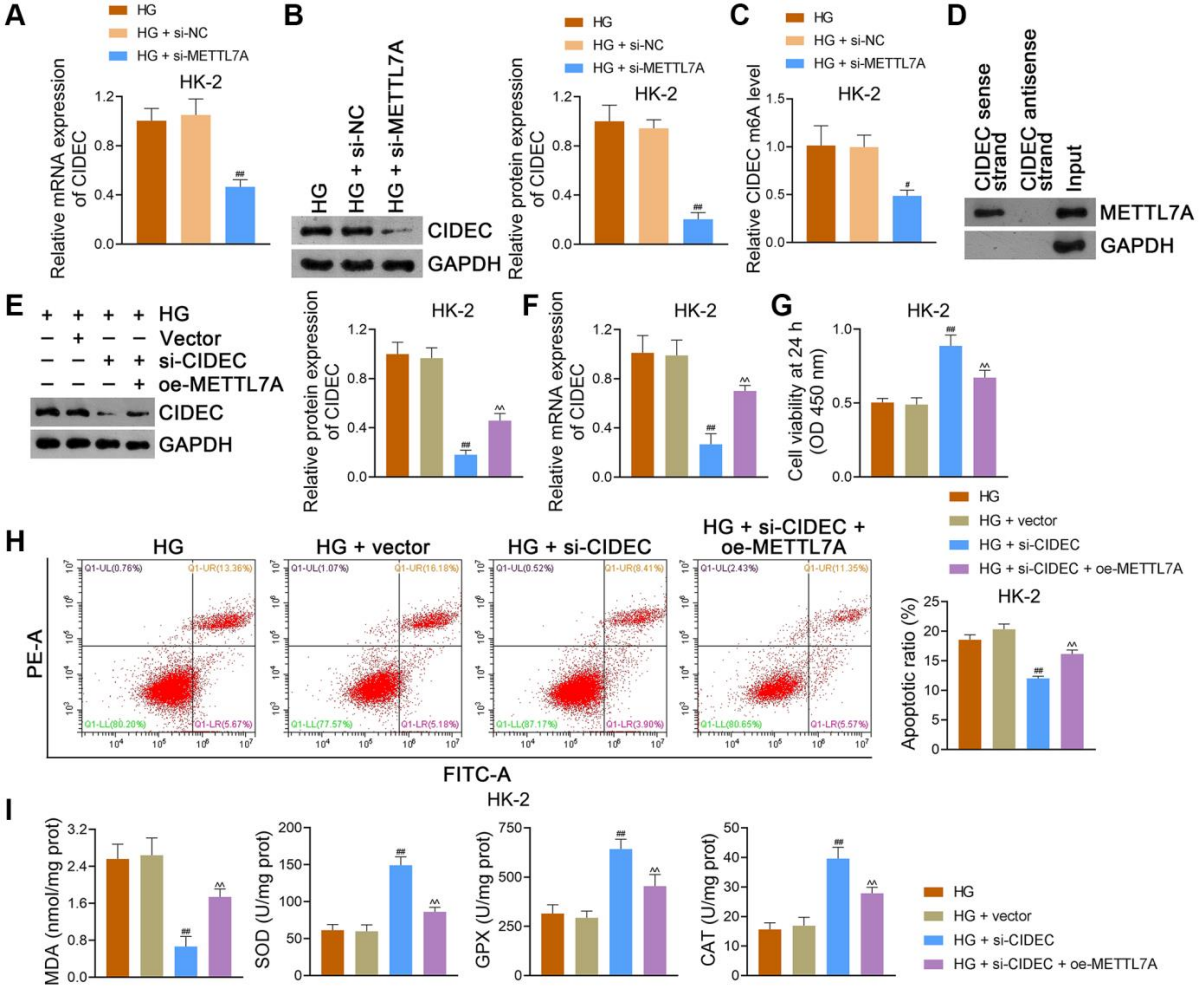
**METTL7A silencing prevents DN progression by downregulating CIDEC m6A methylation in HG-induced HK-2 cells.** (**A**, **B**) The expression of CIDEC in HK-2 cells using qRT-PCR and western blotting. (**C**) CIDEC m6A methylation level in HK-2 cells using MeRIP-qPCR. (**A**–**C**) Data were expressed as mean ± standard deviation. ^#^*p* < 0.05 and ^##^*p* < 0.01 vs. HG + si-NC group. (**A**–**C**) HG-induced HK-2 cells were partially transfected with si-METTL7A or si-NC (negative control) for 48 h to explore the effects of METTL7A on CIDEC and its m6A methylation. (**D**) Determination of the interaction between METTL7A and CIDEC in HEK293T cells using RNA pulldown assay. (**E**, **F**) The expression of CIDEC in HK-2 cells using western blotting and qRT-PCR. (**G**, **H**) HK-2 cell viability and apoptosis using CCK-8 and flow cytometry, respectively. (**I**) The levels of MDA, SOD, GPX, and CAT in HK-2 cells using ELISA. (**E**–**I**) Empty vector (control), si-CIDEC, or/and oe-METTL7A were transfected into HG-induced HK-2 cells to further determine the interaction between METTL7A and CIDEC in DN. Data were expressed as mean ± standard deviation. ^##^*p* < 0.01 vs. HG + vector group; ^^^^*p* < 0.01 vs. HG + si-CIDEC group. Abbreviations: METTL: methyltransferase-like; DN: diabetic nephropathy; CIDEC: cell death-inducing DFF45-like effector C; m6A: N6-methyladenosine; HG: high glucose; qRT-PCR: quantitative real-time polymerase chain reaction; MeRIP-qPCR: methylated RNA immunoprecipitation-PCR; CCK-8: cell counting kit-8; MDA: malondialdehyde; SOD: superoxide dismutase; GPX: glutathione peroxidase; CAT: catalase; ELISA: enzyme-linked immunosorbent assay.

CIDEC is a key factor in insulin resistance, and its silencing has been linked to the prevention of DN progression [31]. To determine whether there is an interaction between METTL7A and CIDEC in DN, HG-induced HK-2 cells were transfected with si-METTL7A. In the present study, CIDEC expression was notably reduced by si-METTL7A in HK-2 cells (*p* < 0.01; Figure 8A, 8B). After si-METTL7A transfection, the CIDEC m6A level in HK-2 cells was significantly lower than that in si-NC-transfected cells (*p* < 0.05; Figure 8C). Similar results were obtained in NRK-52E cells (Supplementary Figure 4A–4C). Through RNA pulldown assay, METTL7A was identified in the RNA CIDEC-protein complex in HEK293T cells, further indicating the interaction between METTL7A and CIDEC (Figure 8D).

Additionally, HG-induced HK-2 cells were transfected with si-CIDEC and/or oe-METTL7A to further explore the regulatory relationship between METTL7A and CIDEC during DN development. Our data revealed that si-CIDEC markedly reduced CIDEC expression in HK-2 cells (*p* < 0.01), which was reversed by oe-METTL7A transfection (*p* < 0.01; Figure 8E, 8F). After si-CIDEC transfection, the viability of HK-2 cells was significantly increased, and apoptosis was inhibited compared to that of empty vector-transfected cells (*p* < 0.01; Figure 8G, 8H). Nevertheless, oe-METTL7A transfection undid the viability-promotive and apoptosis-suppressive effects of si-CIDEC on HK-2 cells (*p* < 0.01; Figure 8G, 8H). Moreover, MDA levels in HK-2 cells were markedly decreased, and the levels of SOD, GPX, and CAT were increased following si-CIDEC transfection (*p <* 0.01), which was abolished by oe-METTL7A transfection (*p* < 0.01; Figure 8I). Consistent findings were obtained in NRK-52E cells (Supplementary Figure 4D–4H).

### ADSC-derived exosomal miR-204 ameliorates DN symptoms by suppressing CIDEC m6A methylation *in vivo*

**Figure 9:**
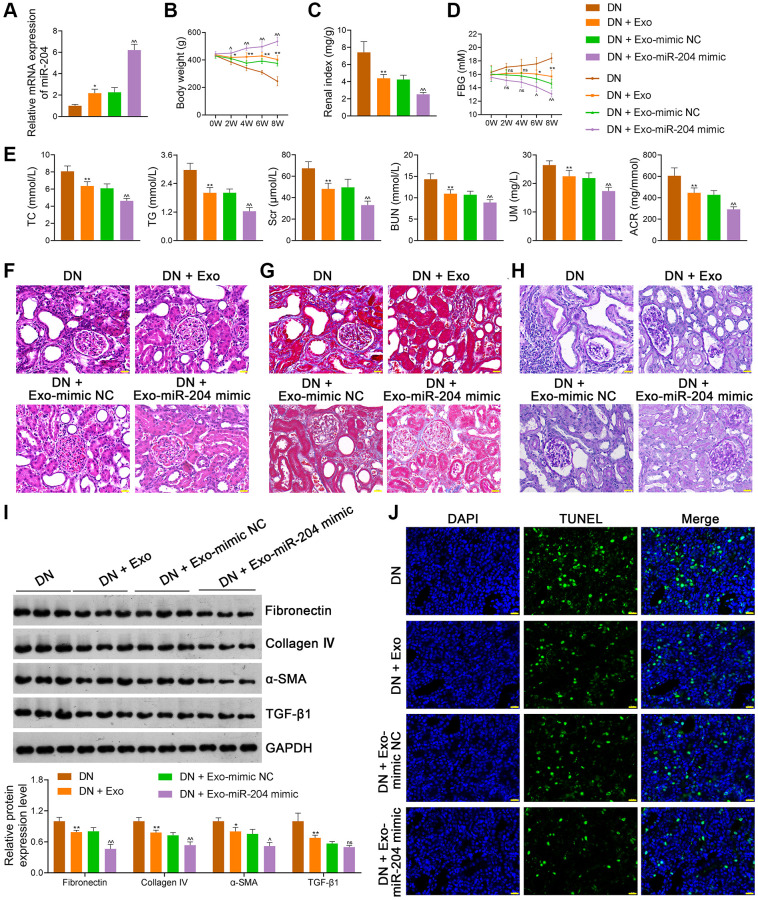
**ADSC-derived Exos loaded with miR-204 ameliorate the symptoms of DN rats.** (**A**) The expression of miR-204 of rats in each group using qRT-PCR. (**B**–**D**) The body weights, renal index, and FBG levels of rats in each group. (**E**) Plasma TC, TG, Scr, BUN, UM, and ACR of rats in each group using an enzymatic colorimetric assay and ELISA. (**F**–**H**) Histopathological examinations in the kidney tissues of rats in each group using HE, Masson, and PAS staining (scale bar = 20 μm). (**I**) The expression of fibronectin, collagen IV, α-SMA, and TGF-β1 in the kidney tissues of rats in each group using western blotting. (**J**) Cell apoptosis in the kidney tissues of rats in each group using a TUNEL assay (scale bar = 20 μm). Rats in the Exo group were treated with 1.6 mg/kg ADSC-derived Exos via caudal vein injection every two days for eight successive weeks. At the same time, rats in the DN + Exo-mimic NC and DN + Exo-miR-204 mimic groups were treated with ADSC-derived Exos loaded with mimic-NC (negative control) or miR-204 mimic, respectively. Rats in the DN group were injected with the same volume of saline. Data were expressed as mean ± standard deviation (*n* = 6/group). ^*^p < 0.05 and ^**^p < 0.01 vs. DN group; ^^^*p* < 0.05 and ^^^^*p* < 0.01 vs DN + Exo-mimic NC group; ns indicates no significant differences between groups. Abbreviations: ADSC: adipose-derived stem cell; Exos: exosomes; miR-204: microRNA-204; DN: diabetic nephropathy; qRT-PCR: quantitative real-time polymerase chain reaction; FBG: fasting blood-glucose; TC: total cholesterol, TG: triglyceride; BUN: blood urea nitrogen; Scr: serum creatinine; UM: urinary microalbumin; ACR: albumin to creatinine ratio; ELISA: enzyme-linked immunosorbent assay; HE: hematoxylin-eosin; PAS: periodic acid-Schiff; α-SMA: alpha-smooth muscle actin; TGF-β1: transforming growth factor beta 1; TUNEL: terminal deoxynucleotidyl transferase-mediated dUTP nick-end labeling.

Since the *in vitro* experiments demonstrated that ADSC-derived exosomal miR-204 prevented DN progression by inhibiting METTL7A-mediated CIDEC m6A methylation, we performed *in vivo* assays to further verify this mechanism. Our results showed that miR-204 mimic-loaded Exos further enhanced miR-204 expression in DN rats compared to mimic-NC-loaded Exos (*p* < 0.01; Figure 9A). In addition, miR-204 mimic-loaded Exos exhibited a greater ability to increase body weight and decrease the renal index of DN rats than mimic-NC-loaded Exos (*p* < 0.05; Figure 9B, 9C). Moreover, miR-204 mimic-Exos exerted a stronger effect on decreasing FBG levels in DN rats than mimic-NC-Exos (*p* < 0.05; Figure 9D). Similarly, the levels of TC, TG, Scr, BUN, UM, and ACR in DN rats were further reduced by miR-204 mimic-loaded Exos compared to mimic-NC-loaded Exo treatment (*p* < 0.01; Figure 9E). We found that miR-204 mimic-loaded Exos further alleviated the histopathological injury (Figure 9F–9H) and downregulated the expression levels of fibronectin, collagen IV, and α-SMA in DN rats when compared to mimic NC-Exos (*p* < 0.05; Figure 9I). Moreover, miR-204 mimic-loaded Exos more effectively inhibited cell apoptosis in the kidney tissues of DN rats than mimic-NC-loaded Exos (Figure 9J).

**Figure 10:**
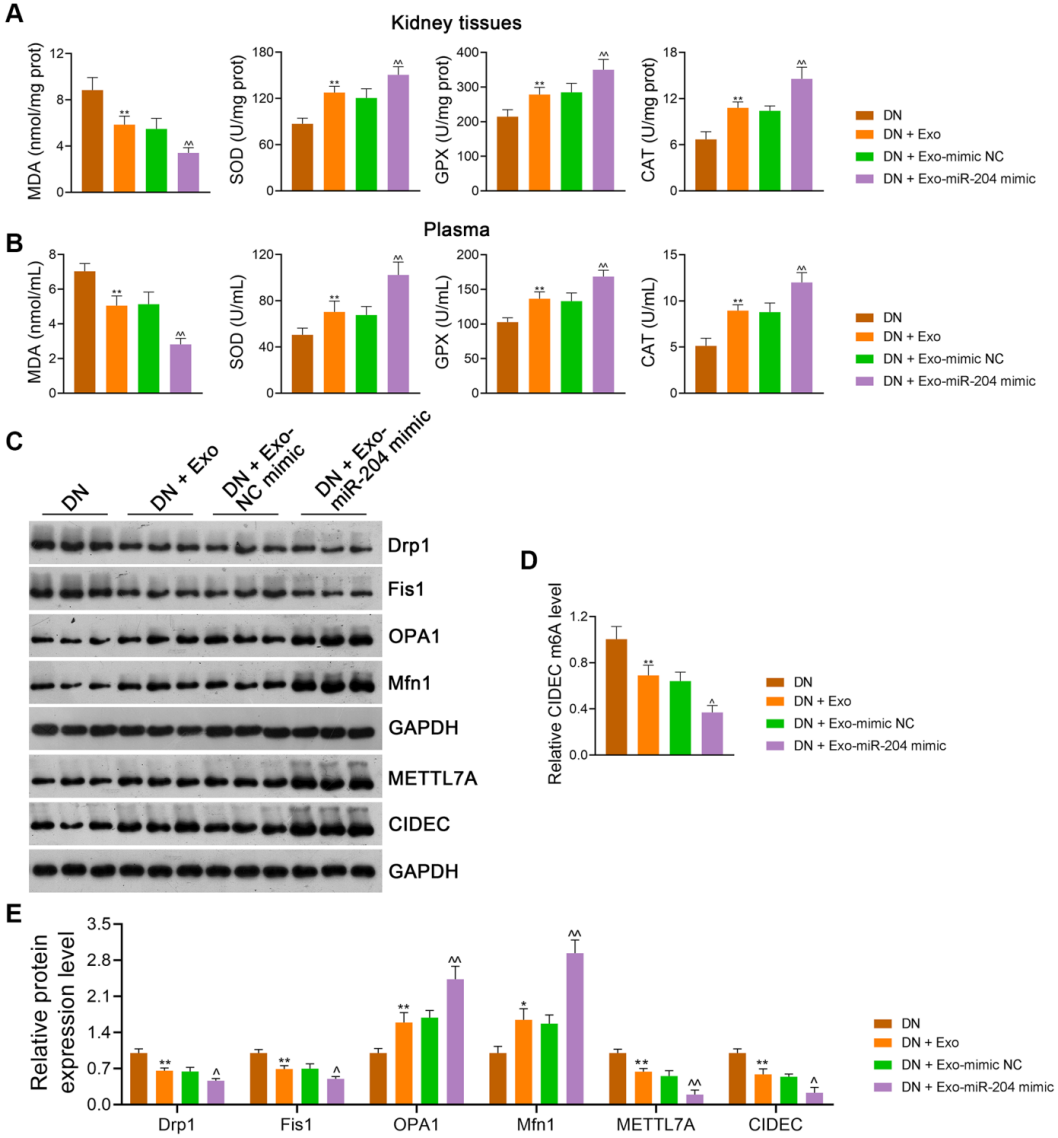
**ADSC-derived Exos modified by miR-204 rescue OS-induced mitochondrial dysfunction in DN rats by inhibiting METTL7A-mediated CIDEC m6A methylation.** (**A**, **B**) The levels of MDA, SOD, GPX, and CAT in the kidney tissues and plasma of rats in each group using ELISA. (**C**, **D**) The expression of Drp1, Fis1, OPA1, Mfn1, METTL7A, and CIDEC in the kidney tissues of rats in each group using western blotting. (**E**) CIDEC m6A methylation levels in HK-2 cells using MeRIP-qPCR. Rats in the Exo group were treated with 1.6 mg/kg ADSC-derived Exos via caudal vein injection every two days for eight successive weeks. Meanwhile, rats in the Exo-mimic NC and Exo-miR-204 mimic groups were treated with ADSC-derived Exos loaded with mimic-NC or miR-204 mimic, respectively. Rats in the DN group were injected with the same volume of saline. Data were expressed as mean ± standard deviation (*n* = 6/group). ^*^p < 0.05 and ^**^p < 0.01 vs. Control group; ^^^*p* < 0.05 and ^^^^*p* < 0.01 vs. DN + Exo-mimic NC group. Abbreviations: ADSC: adipose-derived stem cell; Exos: exosomes; miR-204: microRNA-204; OS: oxidative stress; DN: diabetic nephropathy; METTL: methyltransferase-like; CIDEC: cell death-inducing DFF45-like effector C; m6A: N6-methyladenosine; MDA: malondialdehyde; SOD: superoxide dismutase; GPX: glutathione peroxidase; CAT: catalase; ELISA: enzyme-linked immunosorbent assay; MeRIP-qPCR: methylated RNA immunoprecipitation-PCR.

Compared with mimic-NC-loaded Exos, miR-204 mimic-loaded Exos further downregulated MDA levels and upregulated SOD, GPX, and CAT levels in the kidney tissues (*p* < 0.01; Figure 10A) and plasma (*p* < 0.01; Figure 10B) of DN rats. In addition, miR-204 mimic-loaded Exos exhibited a greater ability to rescue mitochondrial dysfunction than mimic NC-loaded Exos, as the levels of Drp1 and Fis1 in DN rats were reduced and those of OPA1 and Mfn1 were elevated (*p* < 0.05; Figure 10C, 10D). Meanwhile, METTL7A expression in DN rats was further decreased by miR-204 mimic-loaded Exos compared to mimic-NC-loaded Exos (*p* < 0.01; Figure 10C, 10D). In addition, miR-204 mimic-loaded Exos exerted stronger effects on the downregulation of CIDEC expression and m6A levels in DN rats than mimic NC-loaded Exos (*p* < 0.05; Figure 10C–10E).

## DISCUSSION

DN is a prevalent microvascular complication with a high occurrence rate among diabetic patients, leading to increased mortality [35]. Several studies have shown that ADSC-derived Exos can suppress DN progression [4, 16]. In this study, we found that ADSC-derived Exos vividly suppressed OS-induced mitochondrial dysfunction to alleviate the pathological injury of DN. This suggests the potential of ADSC-derived Exos as a novel therapy for DN. Mechanistically, ADSC-derived Exos upregulated miR-204 expression in DN, which then inhibited METTL7A-mediated CIDEC m6A methylation.

Accumulating evidence has demonstrated that OS is the core mechanism implicated in the pathological process of DN [36, 37]. OS is generally defined as an imbalance between pro-oxidants and antioxidants that leads to ROS production [38]. The lipid peroxide, MDA, is the result of ROS interacting with macromolecules and usually serves as a biomarker of OS and tissue oxidative damage [39, 40]. SOD, GPX, and CAT are vital antioxidant enzymes that comprise the pivotal cellular defense mechanism against excessive free radicals [41]. Here, renal ROS and MDA levels in the kidney tissues and plasma of DN rats were upregulated compared to control rats, while SOD, GPX, and CAT levels were downregulated. Notably, we found that the OS inhibitor NAC rescued the symptoms of DN in model rats, which were consistent with the previous study [8].

To our knowledge, mitochondria are the main producers of ROS. Appropriate mitochondrial ROS levels are crucial for cellular homeostasis and signal transduction; however, excessive mitochondrial ROS accumulation can cause damage to cells [42]. Recently, mitochondrial dysfunction has been demonstrated to contribute to the onset and progression of DN [43]. Morphologically, mitochondria are tube-shaped due to their fission-fusion balance [44]. Several fission factors (e.g., Drp1 and Fis1) and fusion factors (e.g., OPA1 and Mfn1) determine the balance between mitochondrial fusion and fission to preserve mitochondrial homeostasis [45]. In particular, excess mitochondrial fission is associated with mitochondrial dysfunction and intracellular ROS generation, which can induce renal tubular cell damage [42]. Functionally, mitochondria mainly act as power factories for cells, producing energy in the form of ATP through a battery of redox reactions [46]. Here, we observed mitochondria damage in DN rats compared to control rats, as well as decreased ATP levels and increased mitochondrial ROS production. Moreover, Drp1 and Fis1 protein levels were upregulated in DN rats, whereas OPA1 and Mfn1 protein levels were downregulated. A prior study showed damaged mitochondria, imbalanced fission and fusion, and excessive ROS production in DN rats [42], which conformed to our observations. Interestingly, we found that NAC treatment exerted a protective effect on the mitochondrial function of DN rats. Collectively, these results demonstrate that OS might induce mitochondrial dysfunction in DN. Therefore, we investigated the role of ADSC-derived Exos on renal mitochondrial function. Consistent with previous findings [4], our results demonstrated that ADSC-derived Exos effectively ameliorated DN symptoms and inhibited cell apoptosis. Significantly, we observed that ADSC-derived Exos suppressed OS and rescued mitochondrial dysfunction in DN rats. These findings suggest that ADSC-derived Exos alleviate DN by suppressing OS-induced mitochondrial dysfunction.

Subsequently, we explored the specific mechanisms of ADSC-derived Exos rescuing mitochondrial dysfunction in DN. Accumulating evidence has shown that the miRNAs secreted by ADSC-derived Exos can attenuate DN pathology [4, 16]. Interestingly, a recent study has revealed that increased miR-204 expression inhibited OS and ameliorated the symptoms of DN [18]. This prompted us to explore the expression pattern of miR-204 in ADSC-derived Exos and its role in DN. Here, we found that miR-204 was highly expressed in ADSC-derived Exos compared with control cells, Significantly, miR-204 upregulation strengthened the suppressive effects of ADSC-derived Exos on pathological injury, OS, and mitochondrial dysfunction in the *in vitro* model of DN. In line with our results, previous studies revealed the beneficial effects of miR-204 in suppressing OS and preventing renal injury in DN [18, 19]. Taken together, these data indicate that ADSC-derived exosomal miR-204 may serve as a novel therapeutic target for DN.

As one of the most common mRNA modifications, m6A methylation acts as a switch in mRNA splicing, expression, translation, and multiple cellular activities [47]. Recent studies have underscored the crosstalk between m6A methylation and miRNAs [26, 27]. Furthermore, increasing evidence indicates the involvement of m6A modification in DN pathogenesis [24, 48]. However, the status of m6A modifications and underlying mechanisms in DN remains largely unclear. In this study, we found increased overall m6A methylation in DN cells compared to control cells. With the advancement of testing techniques and technologies, various m6A modification enzymes have been detected, promoting the understanding of their underlying biological functions [24]. METTL3 and METTL14 are the most studied m6A methyltransferases; however, a recent investigation highlights the m6A methyltransferase activity of METTL7A [25]. Our data showed that METTL7A expression was increased in DN cells compared to control cells. Furthermore, ADSC-derived Exos reduced METTL7A expression in DN cells, and this decrease was intensified by miR-204 upregulation. Using the dual-luciferase reporter assay, we further observed an interaction between miR-204 and METTL7A. Additional *in vitro* experiments revealed that METTL7A overexpression abolished the alleviating effects of miR-204 upregulation on renal tubular cell injury and OS. Taken together, these results suggest that ADSC-derived exosomal miR-204 ameliorates DN symptoms by reducing METTL7A expression. Hence, we further explored the downstream mechanism underlying METTL7A downregulation.

CIDEC, known as a potent apoptotic inducer, has been demonstrated to contribute to DN progression [30, 31]. CIDEC can be found in various tissues and has been indicated to be closely related to the development of metabolic disorders, such as obesity and diabetes [30]. Although the association between METTL7A and CIDEC remains relatively unclear, evidence indicates that METTL7A also functions as a crucial regulator of cell apoptosis [32]. Our results showed that METTL7A silencing downregulated CIDEC expression and m6A methylation. The RNA pull-down assay further demonstrated the interplay between METTL7A and CIDEC. Significantly, we found that CIDEC silencing enhanced renal tubular cell viability, suppressed cell apoptosis, and inhibited OS. A previous study demonstrated that FSP27 (the human congener of CIDEC) could promote OS and cause hepatic damage [49]. Consistent with our findings, another prior study revealed that CIDEC silencing could prevent DN progression by suppressing apoptosis [31]. Moreover, our data showed that METTL7A upregulation abolished the effects of CIDEC silencing on promoting cell survival and suppressing OS in DN. Contrary to our findings, a previous investigation suggested that METTL7A could facilitate pro-survival signaling in choriocarcinoma [32]. This demonstrated the different roles of METTL7A in different pathological conditions. Overall, we perceived that miR-204 might inhibit METTL7A-mediated CIDEC m6A methylation to alleviate renal injury in DN.

Finally, we conducted *in vivo* experiments for further validation. As a result, ADSC-derived Exos ameliorated DN symptoms, inhibited renal cell apoptosis, suppressed OS, and rescued mitochondrial dysfunction in the model rats. Meanwhile, miR-204 expression was elevated and CIDEC m6A methylation levels were reduced by ADSC-derived Exos. Furthermore, the miR-204 upregulation strengthened the alleviating effects of ADSC-derived Exos on DN. These results were in line with the *in vitro* assays, further confirming the therapeutic role and mechanism of ADSC-derived exosomal miR-204 in DN.

This study has some limitations. Firstly, the reliance on animal and cell models raises questions about the applicability of the findings to human DN, due to differences in disease complexity and genetic variability. This gap underscores the need for caution in extrapolating these results to clinical settings. Secondly, the study did further explore the optimal Exo dosage and delivery methods for miR-204, which are critical for ensuring therapeutic efficacy. Finally, the complexity of miRNA mechanisms, particularly the broader biological impacts of miR-204 beyond the METTL7A interaction, is not fully explored. These limitations collectively highlight the need for additional research, including human clinical trials, to validate the effectiveness and safety of ADSC-derived exosomal miR-204 in DN treatment.

## CONCLUSIONS

**Figure 11:**
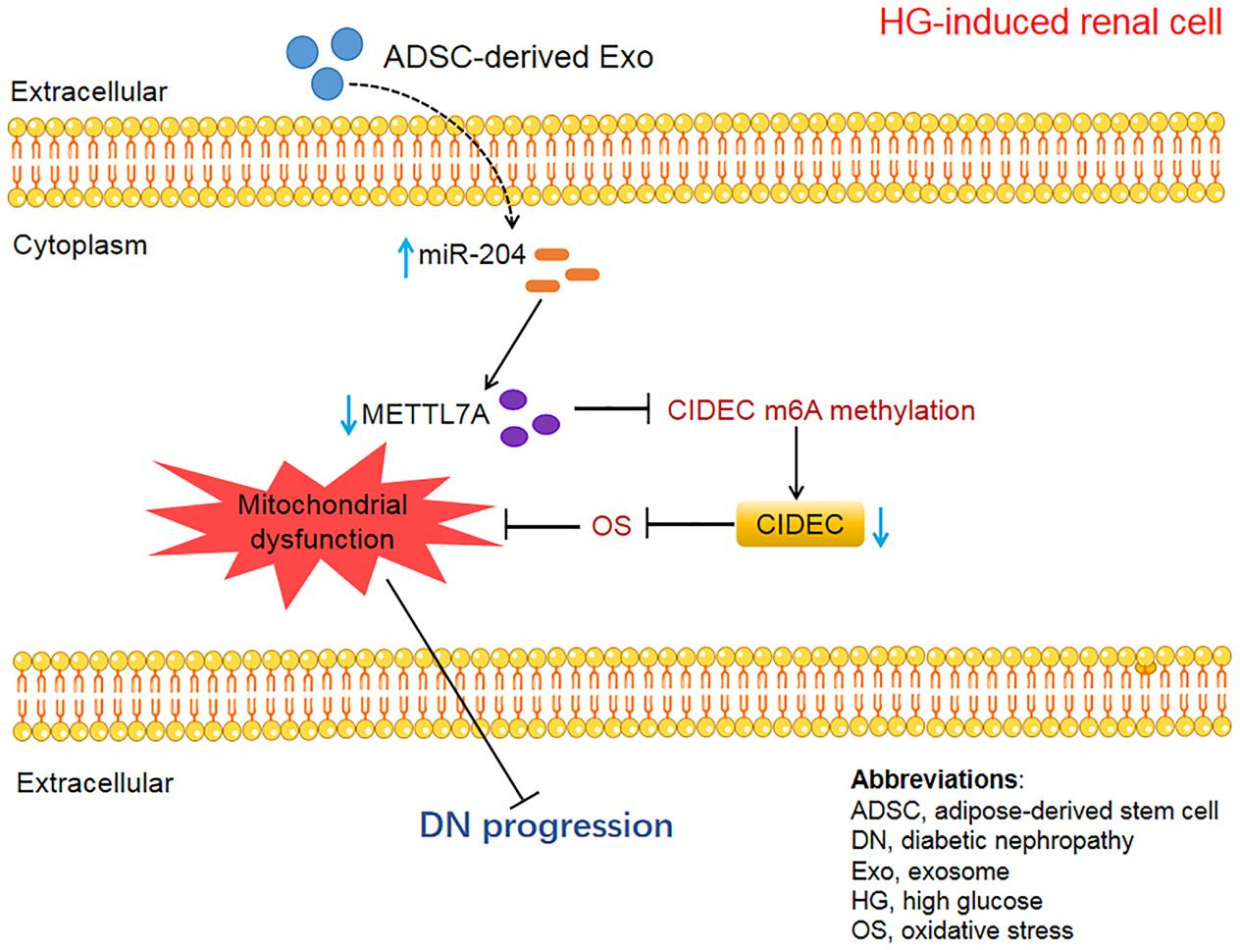
The schematic diagram of the present study.

This study demonstrated that miR-204-modified ADSC-derived Exos rescued OS-induced mitochondrial dysfunction to mitigate DN. This was realized partly through the inhibition of METTL7A-mediated CIDEC m6A methylation. The mechanism through which ADSC-derived Exos alleviate DN was displayed in Figure 11. Building on these findings, future research should aim to validate the clinical effectiveness of ADSC-derived exosomes in treating DN. Further exploration into therapies targeting miR-204, METTL7A, and CIDEC is also warranted. Overall, this study provides novel insights into the molecular mechanisms of DN pathology and directions for follow-up clinical trials, which is beneficial for the improvement of the clinical outcomes of DN patients.

## MATERIALS AND METHODS

### Animal modeling, grouping, and treatments

Fifty-four male Sprague-Dawley rats (specific pathogen-free, aged 8 weeks, 200–250 g) were supplied by the Institute of Comparative Medicine, Yangzhou University, China. All rats were housed in cages and allowed free access to water and food under the following conditions: a 12-h light/dark cycle; 22°C; 35–55% humidity.

### Experiment 1

Eighteen rats were allocated to Control, DN, and DN + NAC (an inhibitor of ROS) groups (*n* = 6/group) at random. Rats in the DN and DN + NAC groups were subjected to high-fat diets (45% kcal fat), whereas control rats were normally bred with 10% kcal fat. After 4 weeks, the rats in DN and DN + NAC groups received an intraperitoneal injection with 35 mg/kg STZ (#S0130, Sigma-Aldrich, St. Louis, MO, USA) dissolving in 1 M sodium citrate (pH 4.5) to induce an *in vivo* model of DN. Rats in the DN + NAC group received an intraperitoneal injection with 20 mg/kg NAC for eight successive weeks. Meanwhile, rats in the control group were administered an equal volume of saline.

### Experiment 2

Twelve rats were randomly divided into DN + PBS and DN + Exo groups (*n* = 6/group). All rats were subjected to high-fat diets (45% kcal fat) for six weeks, followed by an intraperitoneal injection with 35 mg/kg STZ dissolving in 1 M sodium citrate (pH 4.5). After four days, rats were subjected to caudal vein injection with either 1.6 mg/kg PKH26 labeled-Exos or PBS for eight successive weeks.

### Experiment 3

Twenty-four rats were randomly assigned to DN, DN + Exo, DN + Exo-mimic NC, and DN + Exo-miR-204 mimic groups (*n* = 6/group). All rats were fed a high-fat diet (containing 45% kcal fat) for six successive weeks. Subsequently, rats were intraperitoneally injected with 35 mg/kg STZ dissolved in 1 M sodium citrate (pH 4.5). After four days, rats in the Exo group were treated with 1.6 mg/kg ADSC-derived Exos via caudal vein injection every two days for eight successive weeks. Meanwhile, rats in the DN + Exo-mimic NC and DN + Exo-miR-204 mimic groups were treated with ADSC-derived Exos that were loaded with mimic-NC or miR-204 mimic, respectively. miR-204 mimic was used to upregulate miR-204 expression in ADSC-derived Exos, while mimic NC served as the negative control. Rats in the DN group were given a saline injection at the same volume.

Rat body weights and FBG concentrations were monitored every two weeks for two successive months. Blood and urine were collected after 6-h fasting, and all rats were injected with pentobarbital sodium (200 mg/kg) for euthanasia, as previously described [50]. Then, the kidneys were removed and weighed, and the tissues were collected. All collected blood, urine, kidney, and tissue samples were stored at −80°C for later use. Rat renal index was calculated using the following formula: renal index (mg/g) = kidney weight (mg)/body weight (g). The animal experimental procedures were sanctioned by Yangzhou University (202209002).

### Isolation and identification of adipose-derived stem cells (ADSCs)

The adipose tissues were collected and differentiated from the obese rats (The Institute of Comparative Medicine, Yangzhou University, China), which were rinsed twice with cold PBS. Then, adipose tissues were sliced into pieces and digested with 3% type IV collagenase (#17104019; Gibco, Grand Island, NY, USA) for half an hour at 37°C. After collagenase neutralization, the tissues were subjected to centrifugation (1,000 × g) for 10 min at 25°C, and the suspension was filtered through 70-μm screening meshes. ADSCs were obtained by culturing tissues in the Minimum Essential Medium (MEM)-α (#12571071, Gibco) containing 10% fetal bovine serum (FBS; Gibco) with 5% CO_2_ at 37°C. ADSC morphology was photographed under a microscope (BX53; Olympus, Tokyo, Japan).

To induce ADSC differentiation, ADSCs were seeded onto 6-well plates (2 × 10^4^ cells/cm^2^) and cultivated in the Dulbecco’s Modified Eagle’s Medium (DMEM; Gibco) with 5% CO_2_ at 37°C. For adipogenic differentiation, we utilized DMEM consisting of 10% FBS, 3-isobutyl-1-methylxanthine (0.5 mM), dexamethasone (1 μM), insulin (10 μM), and indomethacin (200 μM); an Oil Red O staining kit (#OILR-10001, OriCell, Guangzhou, China) was used to detect differentiated cells. For osteogenic differentiation, we utilized DMEM consisting of 10% FBS, dexamethasone (0.1 μM), ascorbate-2-phosphate (50 μM), and β-glycerophosphate (10 mM); differentiated cells were stained with alizarin red (#ALIR-10001, OriCell). The induction of adipogenic and osteogenic differentiation lasted for 14 and 21 days, respectively. Differentiation was monitored under a microscope (BX53; Olympus).

The surface markers of ADSCs (CD29, CD44, CD45, and CD99) were detected using flow cytometry. Briefly, ADSCs were stained with fluorescein isothiocyanate (FITC) anti-CD29 (#102206, Biolegend, San Diego, CA, USA), FITC anti-CD44 (#163605, Biolegend), FITC anti-CD45 (#157213, Biolegend), and FITC anti-CD99 (#371303, Biolegend) and then incubated for 15 min at 4°C. A flow cytometer (CytoFLEX S, Beckman Coulter, Krefeld, Germany) was employed to analyze the positive cells.

### Extraction and identification of ADSC-derived Exos

ADSCs were seeded onto three plates (10 mm^2^; 5 × 10^6^ cells/plate) and cultivated overnight. Cells were incubated in RPMI 1640 Medium (Gibco) with 10% Exo-free serum for 48 h with 5% CO_2_ at 37°C. The collected cell supernatant was subjected to a series of centrifugation: 2,000 × g, 4°C, 10 min; 2,000 × g, 37°C, 30 min; 10,000 × g, 4°C, 45 min; 100,000 × g, 4°C, 70 min (after filtration through a 0.45-μm membrane); and 100,000 × g, 4°C, 70 min. After supernatant discarding, Exos were suspended in PBS (50 μL). Then, ADSC-derived Exos were collected, which were stored at -80°C till subsequent assays.

ADSC-derived Exos were monitored using a TEM (HT7700, Hitachi), and their sizes were detected using a Flow NanoAnalyzer (NanoFCM, Xiamen, China). ADSC-derived Exos were identified by assessing the expression of Exo markers, CD9, CD63, and CD81, using western blotting.

### Determination of ADSC-derived Exo uptake

To determine whether ADSC-derived Exos can be taken up by rat kidney tissues and HK2 and NRK-52E cells, the PKH67 Green Fluorescent Linker Kit (#MINI67, Sigma-Aldrich) was utilized to label purified ADSC-derived Exos following the user guideline. Next, 1% bovine serum albumin (#A1933, Sigma-Aldrich) was supplemented to bind excess PKH67. For *in vivo* tracking, labeled Exos were washed with PBS, centrifuged, and administered to rats via caudal vein injection. For *in vitro* tracking, HK-2 and NRK-52E cells were cultured together with labeled Exos for 12 h. Cells were stained with 4′,6-diamidino-2-phenylindole (DAPI; #D9542, Sigma-Aldrich), followed by PBS washing and 15 min of fixation in 4% paraformaldehyde. Tissues and cells were observed using a confocal imaging system (UltraVIEW VoX, Perkin Elmer, Waltham, MA, USA).

### Cell culture, transfection, and treatments

Proximal renal tubular epithelial cell lines, HK2 (human) and NRK-52E (rat), as well as human embryonic kidney cell line, HEK293T, were supplied by American Type Culture Collection (Manassas, VA, USA). HK2 and NRK-52E cells were cultivated in 5.5 mmol/L D-glucose DMEM (#21068028, Gibco) comprising penicillin (100 U/mL), streptomycin (100 μg/mL), and 10% FBS (#16140089, Gibco) with 5% CO_2_ at 37°C. HEK293T cells and ADSCs were cultivated in DMEM (1% penicillin-streptomycin; 10% FBS) and MEM-α (10% FBS) with 5% CO_2_ at 37°C, respectively. Cells were incubated in serum-free DMEM for 24 h. To induce *in vitro* models of DN, HK-2 and NRK-52E cells were partially seeded onto DMEM containing 30 mM HG (high glucose) and cultured for another 24 h.

All plasmids used in this study were supplied by GeneChem, Shanghai, China. Small interfering RNAs targeting METTL7A (si-METTL7A) and CIDEC (si-CIDEC) were used to downregulate METTL7A and CIDEC expression, respectively, and METTL7A overexpression (oe-METTL7A) lentiviruses were used to upregulate METTL7A expression. Transfection experiments were conducted using the Lipofectamine 2000 (#11668030, Invitrogen, Carlsbad, CA, USA). Briefly, cells were subjected to 48-h transfection after seeding onto 6-well plates (2 × 10^5^ cells/well). After 6-h incubation at 37°C with 5% CO_2_, cells were cultivated in DMEM (1% penicillin-streptomycin; 10% FBS) for 24 h. We employed qRT-PCR and western blotting to evaluate the effectiveness of transfection.

### Treatment 1

HK-2 and NRK-52E cells were co-cultured with ADSCs that were pre-treated with 2.5 μM GW4869 (an Exo inhibitor) for 8 h. To determine the function of exosomal miR-204 in DN, ADSCs were transfected with miR-204 mimic or miR NC (negative control) for 48 h. Next, Exos were separated from transfected ADSCs and co-cultured with HK-2 and NRK-52E cells for 12 h at the concentration of 100 μg/mL.

### Treatment 2

For exploration of the correlation between miR-204 and METTL7A, HEK293T cells were subjected to miR-204 mimic, miR-204 inhibitor, mimic NC, or inhibitor NC transfection for 48 h.

### Treatment 3

Exos were isolated from either miR-204 mimic- or miR NC-transfected ADSCs, which were subsequently co-cultured with HG-induced HK-2 and NRK-52E cells for 12 h at the concentration of 100 μg/mL. Additionally, HG-induced HK-2 and NRK-52E cells were partially transected with oe-METTL7A and co-cultured with Exos from miR-204 mimic-transfected ADSCs to ascertain the link between miR-204 and METTL7A in DN.

### Treatment 4

HG-induced HK-2 and NRK-52E cells were partially transfected with si-METTL7A or si-NC (negative control) for 48 h. In addition, empty vector (control), si-CIDEC, or/and oe-METTL7A were transfected into HG-treated HK-2 and NRK-52E cells to further ascertain the interaction between METTL7A and CIDEC in DN.

### Enzymatic colorimetric assay

As previously described [51], the enzymatic colorimetric method was applied to assess plasma TC, TG, BUN, and Scr using the following kits: TC (#E-BC-K109-M, Elabscience, Wuhan, China), TG (#E-BC-K261-M), BUN (#E-BC-K329-S, Elabscience), and creatinine (#E-BC-K188-M, Elabscience). Optical density (OD) values were measured using a microplate reader (SpectraMax M4, Molecular Devices, San Jose, CA, USA) to evaluate TC, TG, BUN, and Scr at 510 nm (both TC and TG), 520 nm and 515 nm, respectively. Quantitative analyses were performed by ImageJ (version 1.8.0, NIH, Bethesda, MD, USA).

### Enzyme-linked immunosorbent assay (ELISA)

ELISA kits were utilized for the assessment of 24-h UM, MDA, SOD, GPX, CAT in rat plasma and kidney tissues, as well as in HK-2 and NRK-52 cells. OD values were acquired to evaluate the levels of UM, MDA, SOD, GPX, and CAT at 450 nm, 532 nm, and 600 nm (both for MDA), 560 nm, 412 nm, and 240 nm, respectively, by a microplate reader (DR-3518G, Hiwell Diatek, Wuxi, China). The following ELISA kits were used: microalbumin (#E-EL-R0025c; Elabscience), MDA (#BC0025; Solarbio, Beijing, China), SOD (#BC0170; Solarbio), GPX (#BC1195; Solarbio), and CAT (#ml024518, Mlbio, Shanghai, China). The urinary albumin-to-creatinine ratio (ACR) was calculated, and quantitative analyses were conducted by ImageJ (version 1.8.0, NIH).

### HE, Masson, and PAS staining

Rat kidney tissues were taken, followed by 24-h fixation in 4% paraformaldehyde. Then, tissues were subjected to dehydration with diverse concentrations of ethanol (50%, 70%, 85%, 95%, and 100%). After paraffin embedding, tissues were sectioned at 5 μm, dewaxed with xylene, and hydrated with ethanol. Tissue sections were stained using HE (#C0105S, Beyotime, Shanghai, China), Masson’s trichrome (#G1340), and PAS (G1280) (Solarbio) staining kits according to the manufacturers’ directions. Sections were photographed by a microscope (BX53; Olympus).

### Immunohistochemistry

An immunohistochemistry assay was applied to assess α-SMA and TGF-β1 expression in rat kidney tissues, as previously described [52, 53]. In brief, tissue sections were paraffin-embedded, dewaxed, and dehydrated. Citrate buffer (0.01 M, pH 6.0) was utilized to retrieve antigens. Sections were subjected to primary antibody incubation overnight at 4°C with anti-α-SMA (1:1,000; #ab124964), and anti-TGF-β1 (1:500; #ab215715) (Abcam, Cambridge, UK) and secondary antibody incubation for 15 min at 37°C with Goat Anti-Rabbit IgG H&L (1:2,000; # ab205718, Abcam). Sections were stained with 3, 3’-diaminobenzidine (DAB; #P0202; Beyotime) and photographed by a microscope (BX53; Olympus).

### Terminal deoxynucleotidyl transferase-mediated dUTP nick-end labeling (TUNEL) assay

TUNEL assay was conducted to evaluate apoptotic cells in rat kidney tissues. Paraffin-embedded tissue sections were deparaffinaged, dehydrated, and incubated in a proteinase K solution (50 μL) for 30 min at 37°C. Thereafter, the sections were stained using a TUNEL detection kit (50 μL; Beyotime) at 37°C for 1 h and DAPI for 10 min at 25°C out of the light. Images of apoptotic cells were captured by a confocal imaging system (UltraVIEW VoX; Perkin Elmer).

### qRT-PCR

Total RNA was isolated from ADSC-derived Exos, kidney tissues of rats, HK-2 cells, and NRK-52E cells with TRIzol kits (#15596018, Invitrogen) based on the manufacturer’s directions. To synthesize cDNA, reverse transcription was conducted using the FastKing-RT SuperMix solution (#KR118-02, Tiangen, Beijing, China). A Real-Time PCR Detection System (CFX96 Touch, Bio-Rad, Hercules, CA, USA) and SYBR Green PCR Master Mix (#4364344; Thermo Fisher Scientific, Waltham, MA, USA) were employed for qRT-PCR. The reaction procedure was set as “95°C for 3 min; 95°C for 12 s; 62°C for 40 s (40 cycles)”. The 2^-ΔΔCt^ method was employed for the quantification of gene expression. U6 was used to normalize miR-204 expression, and GAPDH was for the normalization of METTL7A and CIDEC expression. The primers are provided in Table 1.

**Table 1:** Primers used in this study.

**Name**	**Primer sequences (5′ to 3′)**
miR-204 (rat)	Forward: TGGCTACAGTCTTTCTTCA
	Reverse: CTCATGGGACAGTTATGG
miR-204 (human)	Forward: GCGGCGCAAAGAATTCTCCT
	Reverse: GTGCAGGGTCCGAGGT
METTL7A (rat)	Forward: GTGTGCCGAGTGCTGAGG
	Reverse: GGGGGAAGTAAAGGGTGCTC
METTL7A (human)	Forward: TGAACTTTCTGGGCTTGTGGA
	Reverse: ATATGAGGGCGCACCAACTC
CIDEC (human)	Forward: CATGGAGTGAGTGGGACTGG
	Reverse: TATGGGAGAGGGACAGTGGG
CIDEC (rat)	Forward: AGGCTGGGAGGTCTAACACA
	Reverse: TAACACGACAGGGTCTTGCC
GAPDH (rat)	Forward: GCTGAGAATGGGAAGCTGGT
	Reverse: GATGGCATGGACTGTGGTCA
GAPDH (human)	Forward: GGAAAGCCTGCCGGTGACTA
	Reverse: GTGCTAAGCAGTTGGTGGTG
U6 (rat)	Forward: GGGAAACTTCCAGCAAGTCCA
	Reverse: CTCCAGCACGGAAATGCAAG
U6 (human)	Forward: CTCGCTTCGGCAGCACA
	Reverse: AACGCTTCACGAATTTGCGT
si-CIDEC (human)	Sense strand: GGGAGUUUGCAAUAAAUUAUU
	Antisense strand: UAAUUUAUUGCAAACUCCCUA
si-CIDEC (rat)	Sense strand: AGCUGACAAGAAUGGACUAUG
	Antisense strand: UAGUCCAUUCUUGUCAGCUGG

### Western blotting

Separation of total protein from kidney tissues, Exos, as well as HK-2, NRK-52, and HEK293T cells by radioimmunoprecipitation assay (RIPA) lysis buffer (#P0013B, Beyotime). The bicinchoninic acid (BCA) kit (#P0010S, Beyotime) was utilized for protein quantification. Proteins were isolated using 10% sodium dodecyl sulfate-polyacrylamide gel electrophoresis (SDS-PAGE; #P0015A, Beyotime), which was migrated to polyvinylidene difluoride (PVDF) membranes (#FFP24, Beyotime). After 1 h of sealing with 5% nonfat milk (Beyotime), membranes were subjected to primary antibody incubation overnight at 4°C and secondary antibody incubation for 1 h. An enhanced chemiluminescence kit (#G2014, Wuhan, China) was used for the visualization of protein bands, and protein expression was analyzed by ImageJ (version 1.8.0; NIH). In this assay, we used the following antibodies: primary antibodies: anti-fibronectin (1:5,000; #ab45688), anti-collagen IV (1:2,000; #ab182744), anti-Drp1 (1:1,000; #ab184247), anti-Fis1 (1:10,000; #ab156865), anti-OPA1 (1:2,000; #ab157457), anti-Mfn1 (1:1,000; #ab221661), anti-CD9 (1:1,000; #ab236630), anti-CD63 (1:5,000; #ab134045), anti-CD81 (1:5,000; #ab109201), anti-METTL3 (1:1,000, #ab195352), anti-METTL14 (1:1,000; #ab300104), anti-α-SMA (1:20,000; #ab124964), anti-TGF-β1 (1:1,000; #ab215715), anti-GAPDH (1:10,000; #ab181602) (Abcam), anti-METTL7A (1:1,000; #PA5-96971), and anti-CIDEC (1:700; PA1-46128) (Invitrogen, Carlsbad, CA, USA); secondary antibody: Goat anti-rabbit IgG H&L (HRP) (1:2,000, #ab205718, Abcam). Protein expression was quantified by ImageJ (version 1.8.0, NIH) and normalized to GAPDH.

### Detection of tissue ROS and ATP

Detection kits for ROS (DCFHD; #BB-4705, BestBio, Shanghai, China) and ATP (#BC0300, Solarbio) were used to measure the levels of ROS and ATP in the kidney tissues of rats, according to the manufacturers’ directions. For ROS assessment, the fluorescence intensity was monitored at 488 nm (excitation) and 530 nm (emission) using a SpectraMax M4 microplate reader (Molecular Devices, Sunnyvale, CA, USA). Moreover, 100 μL of the tissue supernatant was taken for protein quantification, and the ROS level was calculated as the ratio of fluorescence intensity/protein content (mg). For ATP assessment, the OD values were measured at 340 nm using a NanoDrop 8000 spectrophotometer (ND-8000-GL; Thermo Fisher Scientific).

### TEM observation

Mitochondria were isolated from kidney tissues using a Tissue Mitochondria Isolation Kit (#C3606, Beyotime). The crushed and homogenized kidney tissues were fixed overnight in 2.5% glutaraldehyde (Sigma-Aldrich), followed by treatment with 1% osmium tetroxide for 1 h. After three rounds with PBS, the tissues were embedded in epoxy resin overnight and sliced at 60 nm. The tissue sections were mounted on 200 mesh copper grids and stained with uranyl acetate and citric acid for 10 min. Images of the sections were captured using a TEM (HT7700; Hitachi, Tokyo, Japan).

### Mitochondrial ROS detection

Mitochondrial ROS levels in kidney tissues were measured using the MitoSOX Red dye (#M36008, Invitrogen) based on the manufacturer’s directions. Tissues were stained with the MitoSOX red dye (5 μM) at 37ºC for 10 min. After washing with PBS, the tissues were stained with DAPI for 10 min in the dark and photographed using a confocal imaging system (UltraVIEW VoX, PerkinElmer).

### Cell counting kit-8 (CCK-8) assay

HK-2 and NRK-52E cells were seeded onto 96-well plates (100 μL cell suspension/well) and cultured at 37°C with 5% CO_2_ for 24 h. Then, 10 μL of the CCK-8 kit (#C0037, Beyotime) was added to each well, and cells were incubated for another 2 h. The absorbance at 450 nm was assessed using a microplate reader (DR-3518G, Hiwell Diatek).

### Cell apoptosis assay

HK-2 and NRK-52E cell apoptosis was evaluated using an Annexin V-FITC Cell Apoptosis Detection Kit (#C1062S, Beyotime). After two rounds of PBS washing, cells were suspended with 300 μL of binding buffer. Then, cells were stained with 5 μL of Annexin V-FITC solution and 10 μL of propidium iodide for 15 min and 10 min at 25°C in the dark, respectively. Cell apoptosis was monitored using a CytoFLEX S flow cytometer (Beckman, Miamai, FL, USA) and analyzed using the Cell Quest software (BD Biosciences, Franklin Lakes, NJ, USA).

### m6A RNA methylation detection

The EpiQuik m6A RNA Methylation Quantification Kit (#P-9005-48, 1, Farmingdale, NY, USA) was used to assess the m6A RNA methylation levels in HK-2 and NRK-52E cells, according to the manufacturer’s directions. The absorbance was read at 450 nm on a microplate reader (DR-3518G, Hiwell Diatek) for 2–15 min.

### Dual-luciferase reporter assay

The regulatory relationship between miR-204 and METTL7A was determined using the dual-luciferase reporter assay. Briefly, HEK293T cells were co-transfected with either METTL7A wild-type (METTL7A-WT) or mutant-type (METTL7A-MUT) 3′UTR reporter plasmids and either miR-204 mimic or miR-NC using Lipofectamine 2000. After 48 h of transfection, HEK293T cells were collected and the firefly and Renilla luciferase activities were assessed using a Dual-Luciferase Reporter Assay System (#E1910, Promega, Madison, WI, USA).

### Methylated RNA immunoprecipitation-PCR (MeRIP-qPCR)

MeRIP-qPCR was conducted using a riboMeRIPTM m6A Transcriptome Profiling Kit (#C11051-1, Ribobio, Guangzhou, China) following the manufacturer’s directions. In brief, the RNA concentration was adjusted to 1 μg/μL using Nuclease-free water, and 18 μL of total RNA was added into a 200 μL PCR tube. Then, mRNA fragmentation was performed using an RNA fragmentation reagent (#AM8740, Invitrogen) at 70°C for 7 min. Fragmented mRNA was precipitated with 3 M sodium acetate (#S7899, Sigma-Aldrich) and 20 mg/mL glycogen at -20°C overnight, 10% of which was spared to be utilized as input RNA and stored at −80°C. The prepared MeRIP solution was then added to the anti-m6A magnetic beads. qPCR analysis was applied to evaluate the m6A methylation of CIDEC. The primers used are listed in Table 1.

### RNA pulldown assay

An RNA pulldown assay was performed to determine the interaction between METTL7A and CIDEC in HEK293T cells using the Pierce Magnetic RNA-Protein Pulldown Kit (#20164, Thermo Fisher Scientific) according to the manufacturer’s protocols. CIDEC and CIDEC antisense strands were labeled with RNA biotin, followed by incubation with streptavidin beads at 4°C overnight. After 1 min of centrifugation at 3,000 rpm, the mixture was washed with wash buffer and boiled in loading buffer at 100°C for 10 min. The RNA pulldown complex and METTL7A protein levels were assessed by western blot.

### Statistical analysis

The GraphPad 7.0 software (La Jolla, CA, USA) was employed for statistical analyses. All data are exhibited as mean ± standard deviation. Student’s *t*-test or one-way analysis of variance was applied to evaluate differences between groups. The criterion for statistical significance is *p* < 0.05.

## Supplementary Materials

Supplementary Figures
