# Supplementary material for: Exosomes as nanostructures deliver miR-204 in alleviation of mitochondrial dysfunction in diabetic nephropathy through suppressing methyltransferase-like 7A-mediated CIDEC N6-methyladenosine methylation
Source: Aging (Albany NY). 2024 Feb 8;16(4):3302–31. doi: 10.18632/aging.205535 (PMC10929828; doi:10.18632/aging.205535)
Supplement: Supplementary Figures [file aging-16-205535-s001.pdf]

SUPPLEMENTARY FIGURES

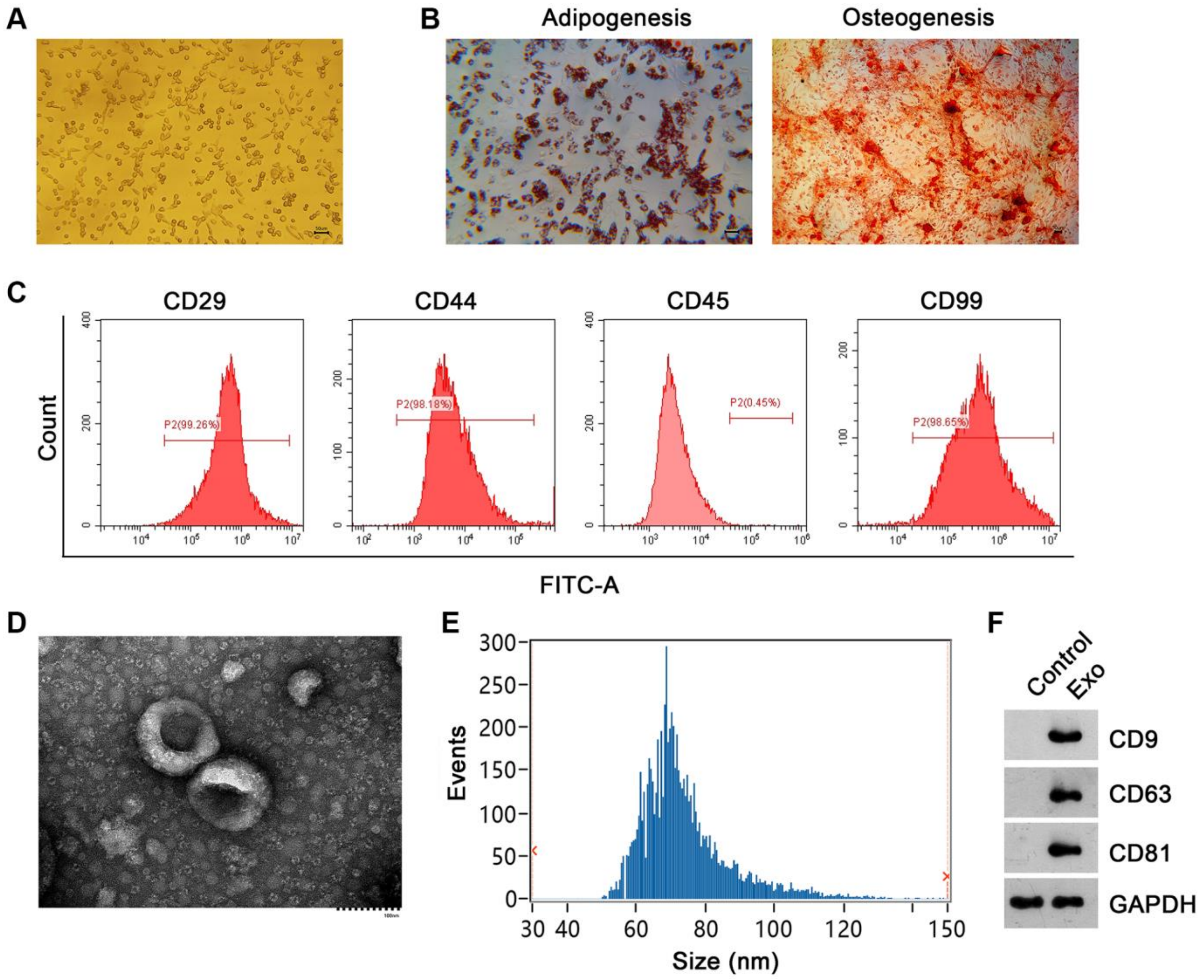

Supplementary Figure 1. Isolation and identification of ADSC-derived Exos. (**A**) The morphology of ADSCs observed under a microscope (scale bar = 50 μm). (**B**) Observation of adipogenic and osteogenic differentiation of ADSCs under a microscope (scale bar = 50 μm). (**C**) The levels of surface markers (CD29, CD44, CD45, and CD99) of ADSCs using flow cytometry. (**D**) TEM showing the morphology of ADSC-derived Exos (scale bar = 100 nm). (**E**) The sizes of ADSC-derived Exos detected using Flow NanoAnalyzer. (**F**) The expression of Exo markers (CD9, CD63, and CD81) using western blotting. Abbreviations: ADSC: adipose-derived stem cell; Exos: exosomes; TEM: transmission electron microscopy.

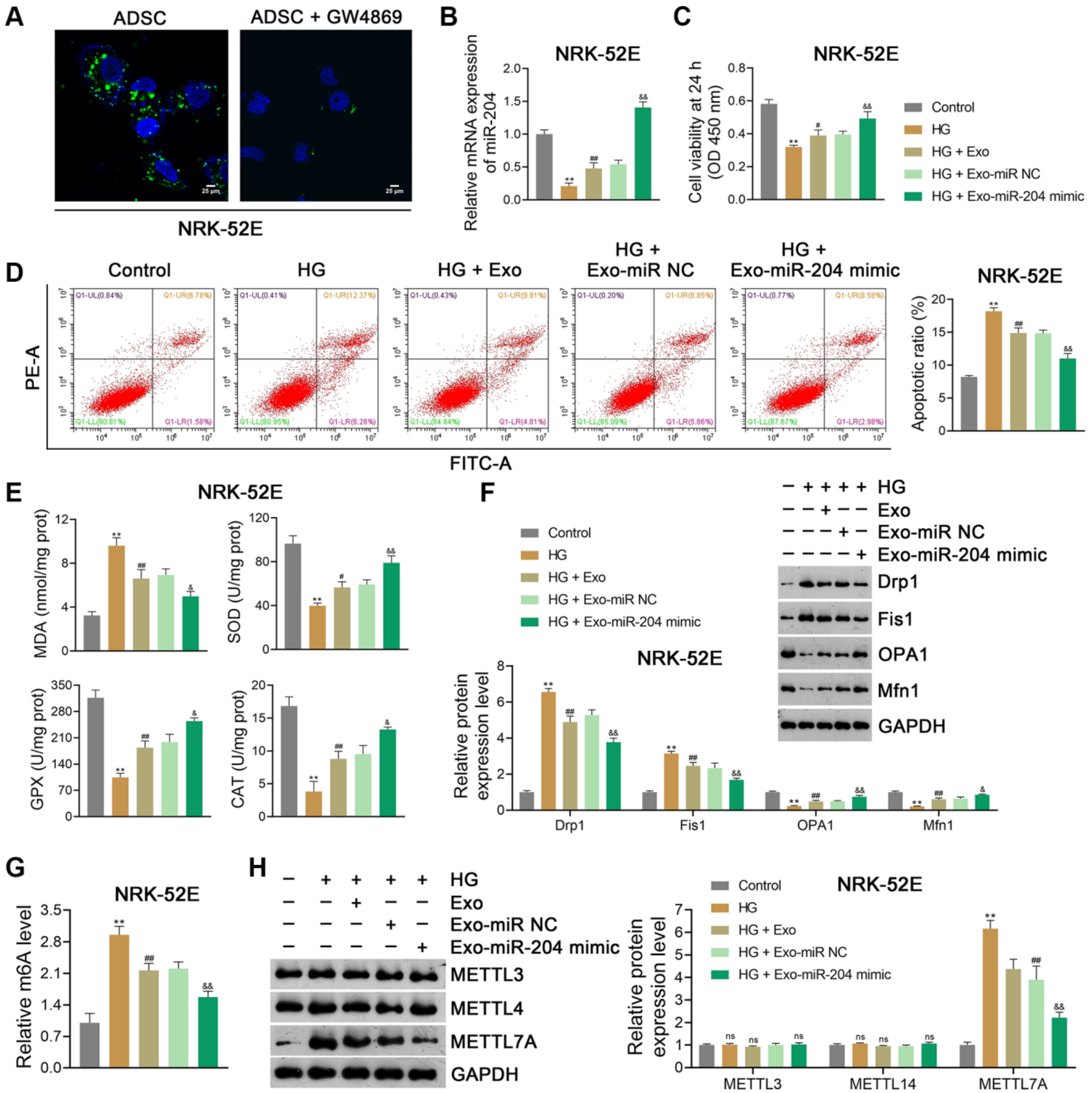

Supplementary Figure 2. ADSC-derived exosomal miR-204 alleviates HG-induced injury and inhibits m6A methylation and METTL7A expression in NRK-52E cells. (**A**) Determination of ADSC-derived Exos taken up by NRK-52E cells (rat proximal renal tubular epithelial cell line) using PKH67 staining. ADSCs were pre-treated with GW4869 (an Exo inhibitor; 2.5 μM), followed by Exo isolation. NRK-52E cells were then co-cultured with PKH67-labeled Exos to evaluate their ability to take up Exos. (**B**) The expression of miR-204 in NRK-52E cells using qRT-PCR. (**C**, **D**) NRK-52E cell viability (at 24 h) and apoptosis using CCK-8 and flow cytometry, respectively. (**E**) Assessment of the levels of MDA, SOD, GPX, and CAT in NRK-52E cells using ELISA. (**F**) The expression of Drp1, Fis1, OPA1, and Mfn1 in NRK-52E cells using western blotting. (**G**) m6A methylation level in NRK-52E cells. (**H**) The expression of METTL3, METTL14, and METTL7A in NRK-52E cells using western blotting. (**B**–**H**) NRK-52E were exposed to HG (30 mM) and cultured for 24 h to construct an *in vitro* model of DN. To determine the function of exosomal miR-204 in DN, ADSCs were transfected with miR-204 mimic or miR NC (negative control) for 48 h. Then, Exos were separated from the transfected ADSCs and co-cultured with NRK-52E cells for 12 h at the concentration of 100 μg/mL. Data were expressed as mean ± standard deviation. $^{**}p < 0.01$ vs. Control group; $^{\#}p < 0.05$ and $^{\#\#}p < 0.01$ vs. HG group; $^{\&}p < 0.05$ and $^{\&\&}p < 0.01$ vs. HG + Exo-miR NC group; ns indicates no significant differences between groups. Abbreviations: ADSC: adipose-derived stem cell; miR-204: microRNA-204; HG: high glucose; m6A: N6-methyladenosine; METTL: methyltransferase-like; Exos: exosomes; qRT-PCR: quantitative real-time polymerase chain reaction; CCK-8: cell counting kit-8; MDA: malondialdehyde; SOD: superoxide dismutase; GPX: glutathione peroxidase; CAT: catalase; ELISA: enzyme-linked immunosorbent assay.

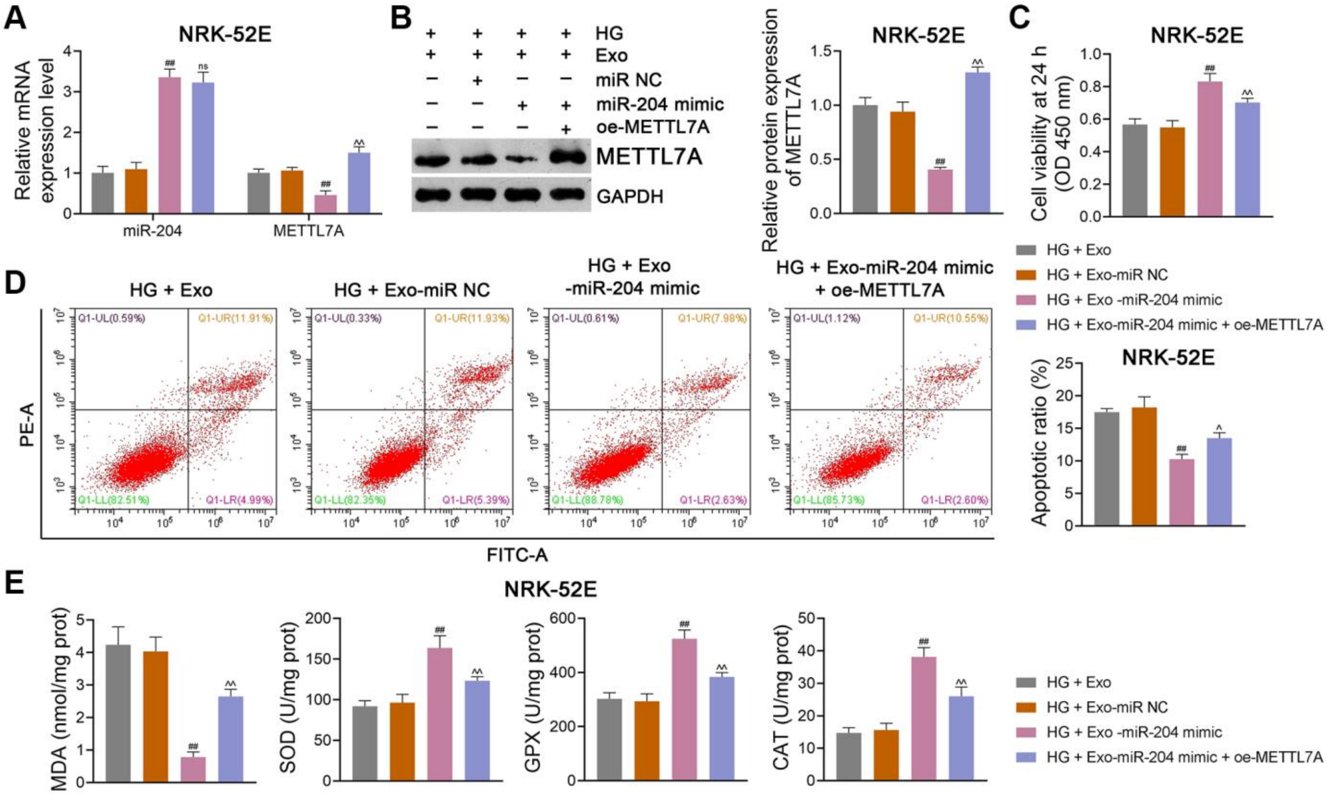

Supplementary Figure 3. ADSC-derived Exos modified by miR-204 prevent DN progression by decreasing METTL7A expression in HG-induced NRK-52E cells. (**A**, **B**) The expression of miR-204 and METTL7A in NRK-52E cells using qRT-PCR and western blotting. (**C**, **D**) NRK-52E cell viability (at 24 h) and apoptosis detected using CCK-8 and flow cytometry, respectively. (**E**) The levels of MDA, SOD, GPX, and CAT in NRK-52E cells using ELISA. Exos were isolated from either miR-204 mimic- or miR NC-transfected ADSCs, which were then co-cultured with HG-induced NRK-52E cells for 12 h at the concentration of 100 μg/mL. Furthermore, HG-induced NRK-52E cells were partially transected with oe-METTL7A and co-cultured with Exos from miR-204 mimic-transfected ADSCs to ascertain the link between miR-204 and METTL7A in DN. Data were expressed as mean ± standard deviation. $^{\#\#}p < 0.01$ vs. HG + Exo-miR NC group; $^{\wedge}p < 0.05$ and $^{\wedge\wedge}p < 0.01$ vs. HG + Exo-miR-204 mimic group; ns indicates no significant differences between groups. Abbreviations: ADSC: adipose-derived stem cell; Exos: exosomes; miR-204: microRNA-204; DN: diabetic nephropathy; METTL: methyltransferase-like; HG: high glucose; qRT-PCR: quantitative real-time polymerase chain reaction; CCK-8: cell counting kit-8; MDA: malondialdehyde; SOD: superoxide dismutase; GPX: glutathione peroxidase; CAT: catalase; ELISA: enzyme-linked immunosorbent assay.

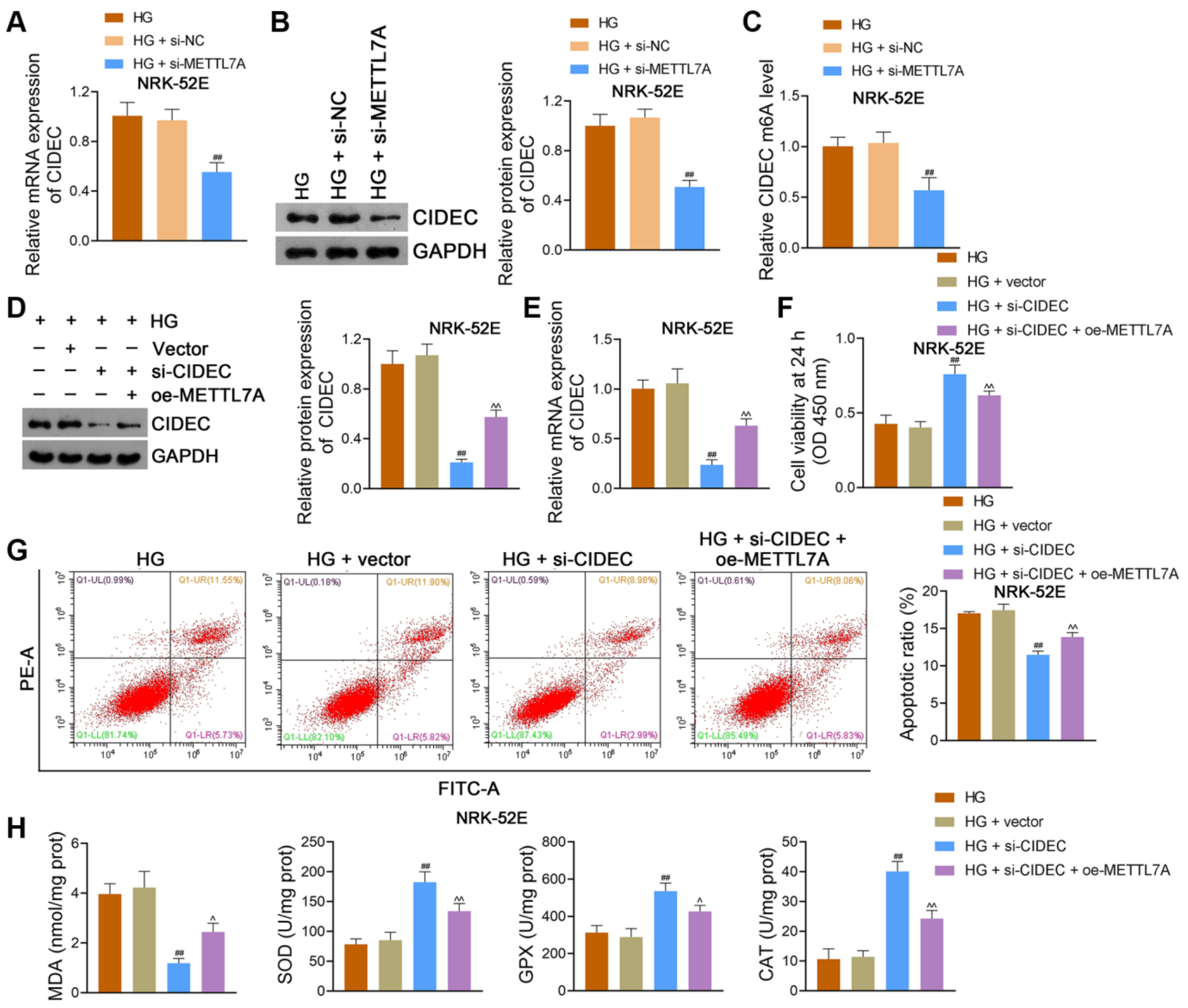

Supplementary Figure 4. METTL7A silencing prevents DN progression by reducing CIDEC m6A methylation in HG-induced NRK-52E cells. (**A**, **B**) The expression of CIDEC in NRK-52E cells using qRT-PCR and western blotting. (**C**) CIDEC m6A methylation levels in NRK-52E cells using MeRIP-qPCR. (**A**–**C**) HG-induced NRK-52E cells were partially transfected with si-METTL7A or si-NC (negative control) for 48 h to explore the effects of METTL7A on CIDEC and its m6A methylation. Data were expressed as mean ± standard deviation. $^{\#\#}p < 0.01$ vs. HG + si-NC group. (**D**, **E**) The expression of CIDEC in NRK-52E cells using western blotting and qRT-PCR. (**F**, **G**) NRK-52E cell viability (at 24 h and apoptosis using CCK-8 and flow cytometry, respectively. (**H**) The levels of MDA, SOD, GPX, and CAT in NRK-52E cells using ELISA. (**D**–**H**) Empty vector (control), si-CIDEC, or/and oe-METTL7A were transfected into HG-induced NRK-52E cells to further ascertain the interaction between METTL7A and CIDEC in DN. Data were expressed as mean ± standard deviation. $^{\#\#}p < 0.01$ vs. HG + vector group; $^{\wedge}p < 0.05$ and $^{\wedge\wedge}p < 0.01$ vs. HG + si-CIDEC group. Abbreviations: METTL: methyltransferase-like; DN: diabetic nephropathy; CIDEC: cell death-inducing DFF45-like effector C; m6A: N6-methyladenosine; HG: high glucose; qRT-PCR: quantitative real-time polymerase chain reaction; MeRIP-qPCR: methylated RNA immunoprecipitation-PCR; CCK-8: cell counting kit-8; MDA: malondialdehyde; SOD: superoxide dismutase; GPX: glutathione peroxidase; CAT: catalase; ELISA: enzyme-linked immunosorbent assay.
